# The efficacy and safety of neoadjuvant chemotherapy on patients with advanced gastric cancer: A multicenter randomized clinical trial

**DOI:** 10.1002/cam4.3224

**Published:** 2020-06-24

**Authors:** Qun Zhao, Changhong Lian, Zhibin Huo, Ming Li, Yang Liu, Liqiao Fan, Bibo Tan, Xuefeng Zhao, Zhidong Zhang, Dong Wang, Yu Liu, Honghai Guo, Peigang Yang, Yuan Tian, Yong Li

**Affiliations:** ^1^ Department of General Surgery The Fourth Hospital of Hebei Medical University Shijiazhuang China; ^2^ Surgical Oncology Changzhi Medical College Affiliated Peace Hospital Changzhi Shanxi China; ^3^ Gastrointestinal Tumor Surgery Xingtai People's Hospital Xingtai China; ^4^ General Surgery The First Hospital of Shijiazhuang Shijiazhuang China

**Keywords:** gastric cancer, neoadjuvant chemotherapy, SOX, survival analysis, XELOX

## Abstract

**Objectives:**

Exploring the efficacy and safety of perioperative chemotherapy on patients with AGC at different clinical and pathological stages.

**Methods:**

A phase III randomized, multicenter, trial comparing adjuvant (arm A) or perioperative S‐1 plus oxaliplatin (SOX, arm B), and perioperative capecitabine plus oxaliplatin (XELOX, arm C) was initiated in T3/4, node + gastric cancer patients (unclear). Each patient received an 8‐cycle chemotherapy (3 weeks for one cycle). Group arms B and C received two cycles preoperatively, and six cycles postoperatively. Primary endpoints were R0 resection rate and DFS, and secondary endpoints included OS, ORR, DCR, and safety. This study was registered on Clinicaltrials.gov. NCT01516944.

**Results:**

A total of 749 patients were randomly assigned into groups A, B, and C. Group A received 1460 circles chemotherapy and group B received 1177 circles while group C received 1200 circles. R0 resection rates in the three groups were 81.7%, 88.7%, and 83.1%, respectively. The difference between groups A and B was considered to be statistically significant (*P* = .018), and no significant difference between groups B and C (*P* = .051). Hazard ratio were compared between groups B and C and DFS showed 0.72 (0.67‐0.77 with 95% CI), *P*_non‐inferiority_ < .0001, *P*_log‐rank_ = .064). The CI top limit actually lower than the estimated value of 1.38, which indicated noninferiority of SOX to XELOX.

**Conclusions:**

Compared with PAC, perioperative chemotherapy showed a significant improvement in R0 resection rates and prognosis in AGC patients with higher safety rates. This study was powered to show superiority of perioperative over adjuvant SOX, and noninferiority of SOX to XELOX. Volume measurement, repeated laparoscopic exploration combined with exfoliative cytology can be used as a supplementary method in the clinical staging and efficacy evaluation of AGC.

## Graphical abstract

This study proved that perioperative chemotherapy showed a significant improvement in R0 resection rates and prognosis in AGC patients with higher safety rates when compared with postoperative adjuvant chemotherapy.

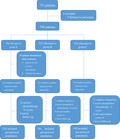

## 1 BACKGROUND

Gastric cancer (GC) is the sixth most common malignance worldwide, and its prevalence is geographically different around the world. According to GLOBOCAN2018, 1.03 million new cases of GC are identified globally each year, 44.13% of which are in China.^1^ The prognosis of GC in China is relatively poor due to lack of gastroscopy screening in some areas of the country. This results in the development of advanced stage cancer, usually with large blood vessel invasion and metastasis to the peritoneum or distant organs often at point of diagnosis. Thus, the outcome from surgery is poor, 5‐year survival rate in patients with advanced gastric cancer (AGCA) receiving radical surgical removal of tumor is only 40%‐50%.^2^ The exploration of an effective clinical treatment plan for patients with AGCA is an important challenge.

The CLASSIC trial confirmed the use of capecitabine plus oxaliplatin (XELOX regimen in postoperative adjuvant therapy for GC. The result showed that the DFS in patients was significantly longer than that in the observation group. Furthermore, the ACTS‐GC trial showed that single‐agent S‐1 adjuvant chemotherapy significantly improves the survival in GC patients. These trials have not only confirmed the effect of postoperative adjuvant chemotherapy (PAC) on the prognosis of patients with GC compared to surgery only, but also have established the use of a chemotherapy regimen based on a combination of platinum and fluorouracil.^3^, ^4^ However, due to the difficulty and risk of surgery in locally advanced patients in stages like IIIC, expansion in surgery does not lead to an improvement in disease cure rate. Even though adjuvant chemotherapy is used in these patients, the rate of recurrence and metastasis still remains high. In recent years, preoperative neoadjuvant chemotherapy (NAC) has been proven effective for patients with locally resectable or potentially resectable AGCA, due to its improvement in R0 resection rate and patient prognosis. Furthermore, this approach is able to test tumor reaction to drug, confirm the necessity for further chemotherapy after surgery, and advice clinicians on treatment plans to improve outcome.

The MAGIC trial is unquestionably a milestone in the development of NAC.^5^ Its result showed that perioperative chemotherapy improved R0 resection rate from 69% to 79% with an increase of 5‐year survival rate by 13%. This research provided evidence for future research on neoadjuvant therapy. Another French clinical study (FFCD9703) observed the use of perioperative cisplatin plus 5‐fluorouracil (5‐FU) compared to surgery alone. The 5‐year disease‐free survival (DFS) rate in perioperative chemotherapy group was 34% compared to 21% in surgery alone group (*P* = .003), suggesting that perioperative FP chemotherapy is able to improve long‐term survival in AGCA. The FLOP4 study is a multicenter open‐label randomized controlled II/III study. Compared with perioperative ECF/ECX treatment (6%), the pathological complete response (CR) rate in FLOT perioperative group was significantly improved (16%).

These studies have become successful precedents for perioperative treatment in locally advanced GC. However, due to the difference in standard protocols for chemotherapy in different countries, a few issues have been brought up. These include the challenge in accurate tumor staging before surgery and objective evaluation of the effectiveness of NAC, and the challenge of giving precise treatment plans to patients with different clinical stages and Lauren classification. Our preliminary study showed that preoperative neoadjuvant therapy using either XELOX^6^ or SOX^7^ decreases cancer stage, increase R0 resection rate and improve overall survival (OS) in patients with AGCA compared to D2 surgery only. To further address the above issues, we have conducted a randomized, controlled, multicenter clinical study comparing the perioperative effect of oxaliplatin with S‐1 or capecitabine (CAP) in the treatment for patients with AGCA.

## 2 METHODS

### 2.1 Study design and participants

Between January 2011 and May 2016, a total of 749 patients from four centers were randomly assigned into groups A (290), B (223), and C (236). All patients were included in the full analysis set (FAS). We defined safety set (SS) in which participants received the designated chemotherapy at least once (243, 223 and 236 patients from groups A‐C, respectively). Two hundred and twenty‐five patients from group A, 201 from group B, and 198 from group C completed all treatment plans. Written informed consents were obtained from all patients. This study is registered with ClinicalTrials.gov, registry number NCT01516944.

### 2.2 Randomization and procedures

All eligible participants were randomized into groups A, B, and C. The randomization is based on the performance status, tumor staging, medical center, and patient health condition.

In group A, patients received D2 gastrectomy without preoperative NAC but received SOX regimen for 2‐6 cycles in 1 month following the surgery (oxaliplatin 130 mg/m^2^ via IV infusion on day 1, S1 40 mg/m^2^, oral bd, postmeal on the 1st to the 14th day). Each cycle lasted for 21 days and repeated 2‐6 circles or the chemotherapy ceases when the participants developed any of the adverse effects which met the exclusion criteria (see the Supplementary Appendix for details about the protocal).

In group B, patients received two cycles of neoadjuvant SOX chemotherapy preoperatively (oxaliplatin 130 mg/m^2^ via IV infusion on day 1, S1 40 mg/m^2^, oral bd, postmeal on the 1st to the 14th day). One month after the chemotherapy, the patients then underwent D2 surgery and then the aforementioned SOX treatment cycles were continued 1 month postoperatively.

In group C, patients received preoperative XELOX regimen for two cycles (oxaliplatin 130 mg/m^2^ via IV infusion on day 1 with CAP 1000 mg/m^2^ oral bd, postmeal on the 1st to the 14th day). Each cycle lasted a period of 21 days. After 1 month of the rest period, radical D2 surgery was scheduled and then the aforementioned XELOX treatment cycles were repeated 1 month postoperatively.

Those patients who accepted the NAC were evaluated with the objective response rate following two cycles, and reassessed with their cancer stage in 4 weeks after the planed treatments were completed. This study mainly investigated surgical R0 resection rates and DFS. This study also evaluated overall response rate (ORR), disease control rate (DCR), and OS, as well as the safety. The advantages and disadvantages among the three chemotherapy regimens were also analyzed.

### 2.3 Sample size estimation

This study was aiming to confirm that the outcome of S1 plus oxaliplatin treatment is better compared with surgery alone (*α* = 0.05, *β* = 0.2) in terms of R0 resection rates and the desired therapeutic effect of the drug is 10%. Oxaliplatin combined with CAP is comparative to S1 plus oxaliplatin. The estimated sample size was 656 cases but in consideration of 10% of exclusion rate, a total of 729 patients were recruited in order to gain an 80% statistical power.

### 2.4 Statistical analysis of data sets

Full analysis set: Based on intention‐to‐treat analysis, all participants were included in this set. If any lack of efficacy remarks, concluded with the previous results.

Safety set: Participants who received at least one dose of the designated chemo drug.

Per‐protocol set: Those patients who meet the eligible criteria and completed all planned treatment, but does not meet the exclusion criteria fell into this set. The designed treatments are suitable for these patients with satisfied compliance and a completed medical record which is consistent with the requirements of case report forms. As a result, group A was treated with gastrectomy followed by a postoperative adjuvant therapy (more than four circles) with completed follow‐ups. Groups B and C received presurgical chemotherapy (≥2 cycles) and gastrectomy followed by treatment of ≥4 cycles of postoperative adjuvant therapy with completed follow‐ups.

### 2.5 CT efficacy evaluation

Based on RECIST 1.1, efficacy was defined as CR, partial response (PR), stable disease (SD), and progressive disease (PD). Complete response in addition to PR represented ORRs, while CR, PR, and SD altogether represented DCRs. If lacking efficacy index occurred, used the previous results based on the intention‐to‐treat concept.

### 2.6 CT TNM staging

Tumor staging was evaluated both before and after NAC, according to the seventh version of GC TNM staging system published by Union for International Cancer Control and American Joint Committee on Cancer TNM staging in 2010.

### 2.7 Adverse reaction evaluation

The Common terminology criteria for adverse event (AE) 4.0 was used to evaluate toxicity ranking from 0 to 4 (grade 0—nil AEs, 1—mild AEs, 2—moderate, 3—severe AEs, 4—life‐threatening or disabling AEs and 5—deaths).

### 2.8 Surgical evaluation

The resection rates were defined as R0, R1, and R2 based on exploratory surgery, surgical records, and postoperative pathological results.

### 2.9 Follow‐up situation

All patients received follow‐ups every third month by written letters, phone interview, clinic visits or readmissions. The total follow‐up period ranged from 3.6 to 28.6 months (median 23.5) and ended by July 1, 2017. Total survival began to be counted from recruitment to death by any causes, while DFS began from the gastrectomy to disease recurrence or death by any causes. For those patients who were not followed up for the disease progress or death, the last time of follow‐up of these patients were used and considered as censored data.

### 2.10 Statistical analyses

Statistical analyses were performed using SPSS 21.0 including FAS, PP, SS. Enumeration data were described as frequency (constituent ratio), while quantitative data were expressed as $\bar{x}$ ± s. Enumeration data among groups were compared with the chi‐squared test. Quantitative data were analyzed with single factor ANOVA. Ranked data were analyzed with the Kruskal‐Wallis *H* test. Survival was estimated with Kaplan‐Meier curves. Survival rates among groups were compared utilizing the log‐rank test. The primary efficacy was estimated by Cox regression hazard ratio (HR) between an 95% CI in terms of the noninferiority of SOX to XELOX by DFS. Log‐rank was used to calculate the HR and 95% confidence interval for each subgroup, using the statistical software stata 15.0 command to generate a forest map. *P* < .05 was considered to be statistical significant.

## 3 RESULTS AND CONCLUSION

**FIGURE 1:**
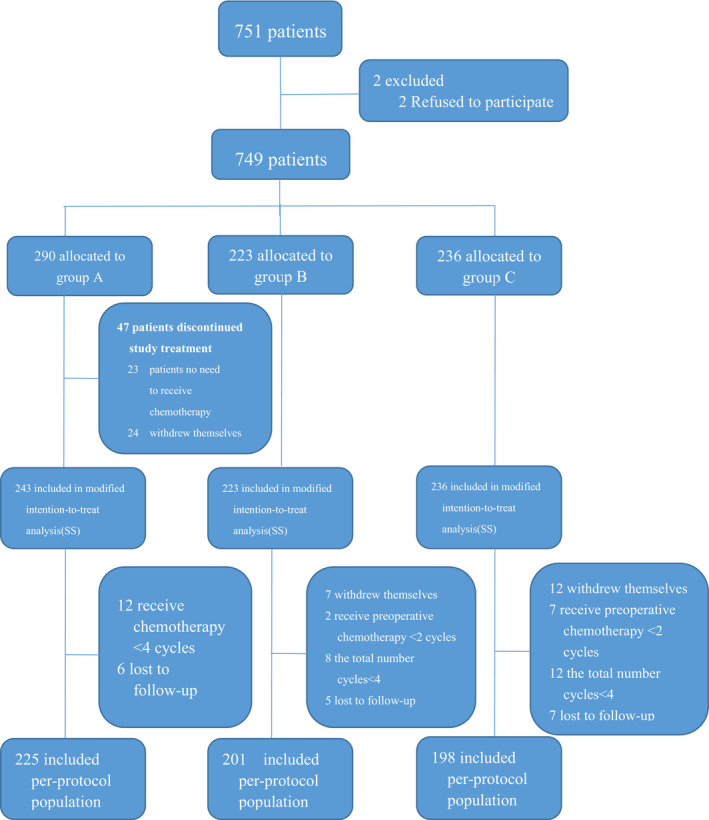
Trial profile

Between January 2011 and May 2016, we randomly allocated a total of 751 AGCA patients from Hebei Medical University, Shanxi Changzhi Peace Hospital, Xingtai City People's Hospital and Shijiazhuang City People's Hospital. Excluding two patients who were not willing to participate in our study, the rest 749 participants were randomly assigned into groups A, B, and C on a 1:1:1 ratio (see the Appendix for details about entry and exclusion criteria). A total of 749 patients were included in the FAS. The SS was defined by who received at least once designated chemotherapy of their group. Therefore, 47 patients in group A who did not undertake PAC were excluded (leaving 243 patients in group A, 223 in B, and 236 in C). At the end, only a number of 225/290 of patients from group A, 201/223 of B, and 198/236 of C completed all protocol treatment, which were included PP (Figure 1). Table 1 represents the Baseline characteristics of the study population in FAS, which are generally similar.

**TABLE 1:** Baseline characteristics (FAS)

	Group A (290)	Group B (223)	Group C (236)	*χ* ^2^	*P*
Gender				2.097	.351
Male	219	177	174		
Female	71	46	62		
Age				4.016	.134
≤60	147	103	99		
＞60	143	120	137		
Tumor position				1.817	.936
Cardiac and gastric fundus	117	98	94		
Gastric body	53	40	42		
Gastric antrum	101	68	81		
Total stomach and multifocal	19	17	19		
Borrmann type				12.112	.060
Borrmann Ⅰ	5	3	3		
Borrmann II	95	97	84		
Borrmann III	178	109	143		
Borrmann IV	12	14	6		
Pathological staging				0.119	.998
High and middle differentiation	134	101	110		
Low differentiation	123	97	99		
Poor differentiation	33	25	27		
Clinical staging				5.534	.699
IIA	39	27	27		
IIB	49	39	40		
IIIA	98	77	67		
IIB	71	50	71		
IIIC	33	30	31		

Abbreviation: FAS, full analysis set.

### 3.1 Comparison of chemotherapy cycle lengths

Chemotherapy cycle lengths were compared using PP. The median length among three group of patients showed no statistical difference (*P *＝ 0.16). The dose strength of the median level of group C was higher than that of group B (Tables 2A,B). Two hundred and twenty‐five patients in group A underwent a total of 1460 cycles with 275 cycles having delays. The main cause of withdrawal or adjustment of the dosage of the chemo drugs is because the participants' condition was deteriorated. There were 113 cycles that had dose adjustment. Two hundred and one patients in group B undertook 1177 cycles with 208 cycles having delays. The most common cause of withdrawal was disease progress.^8^ There were 111 cycles that had dose adjustment.

**TABLE 2A:** Treatment exposure (PP)

	Group A (N = 225)	Group B (N = 201)	*P*
Total number of cycles	1460	1177	
Cycles with delayed schedule	275 (19%)	279 (24%)	.51
Because of neutropenia	76 (28%)	81 (29%)	
Because of leukopenia	69 (25%)	72 (26%)	
cycles with dose modification	113	129	.53
Because of neutropenia	52 (46%)	64 (50%)	
Because of leukopenia	43 (38%)	52 (40%)	
Relative dose intensity			.48
Oxaliplatin (median [%])	127.3 (98%)	118.4 (91%)	
S‐1 or capecitabine (median [%])	78.4 (98%)	75.4 (94%)	

Note

Data are n (％).

**TABLE 2B:** Treatment exposure (PP)

	Group B (N ＝ 201)	Group C (N ＝ 198)	*P*
Total number of cycles	1177	1200	
Cycles with delayed schedule	279 (24%)	208 (17%)	.29
Because of neutropenia	81 (29%)	44 (21%)	
Because of leukopenia	72 (26%)	32 (15%)	
cycles with dose modification	129	111	.41
Because of neutropenia	64 (50%)	51 (46%)	
Because of leukopenia	52 (40%)	46 (41%)	
Relative dose intensity			<.0001
Oxaliplatin (median [%])	118.4 (91%)	126.1 (97)	
S‐1 or capecitabine (median [%])	75.4 (94%)	1925.4 (96%)	

Note

Data are n (％).

#### 3.1.1 Comparison of surgical evaluation

Full analysis set was used for surgical evaluation. All 290 patients in group A undertook surgical treatment along with exploratory surgery and postoperative pathological analysis with 32 of these patients receiving noncurative surgery, 15 undergoing mere exploratory surgery, and 6 receiving gastrojejunostomy. In the group B, 7 out of the total 233 patients did not undertake surgical treatment due to disease progress or other causes. One hundred and ninety‐eight of these 233 patients received resection surgery, while seven of them received noncurative surgery, and 11 underwent mere exploratory surgery. In group C, there were 214 allocated patients. Twelve of these patients did not undertake surgical treatment due to disease progress or other causes. One hundred and ninety‐six of them undertook curative gastrectomy while 19 of them underwent noncurative resection surgery and 6 of them received pure exploratory surgery. Three patients in group C received gastrojejunostomy. The R0 resection rates of A, B, and C groups were 81.7% (237/290), 88.7% (198/223), and 83.1% (196/236), respectively. There were statistical differences in the R0 resection rates between the three groups (*P* < .05; Table 3A).The difference between A and B groups was statistically significant (*P* = .018), while it between B and C and it between A and C was not (*P* = .051 and *P* = .390, respectively) (Table 3B).

**TABLE 3A:** R0 rate of each group (FAS)

	R0	R1/Rx	No. of surgery	R0 rate (%)	*P*
Group A (N = 290)	237	32	21	81.72	.022
Group B (N = 223)	198	7	18	87.79	
Group C (N = 236)	196	19	21	83.05	

Abbreviation: FAS, full analysis set.

**TABLE 3B:** Comparison of R0 rates (FAS)

	Rate difference	Confidence interval	*χ 2*	*P*
Group A vs Group B	0.0607	−0.0278, 0.0547	4.881	.018
Group B vs Group C	−0.0474	−0.0512, 0.0420	3.106	.051
Group A vs Group C	0.0133	0.0124, 0.1032	0.157	.390

Abbreviation: FAS, full analysis set.

**FIGURE 2:**
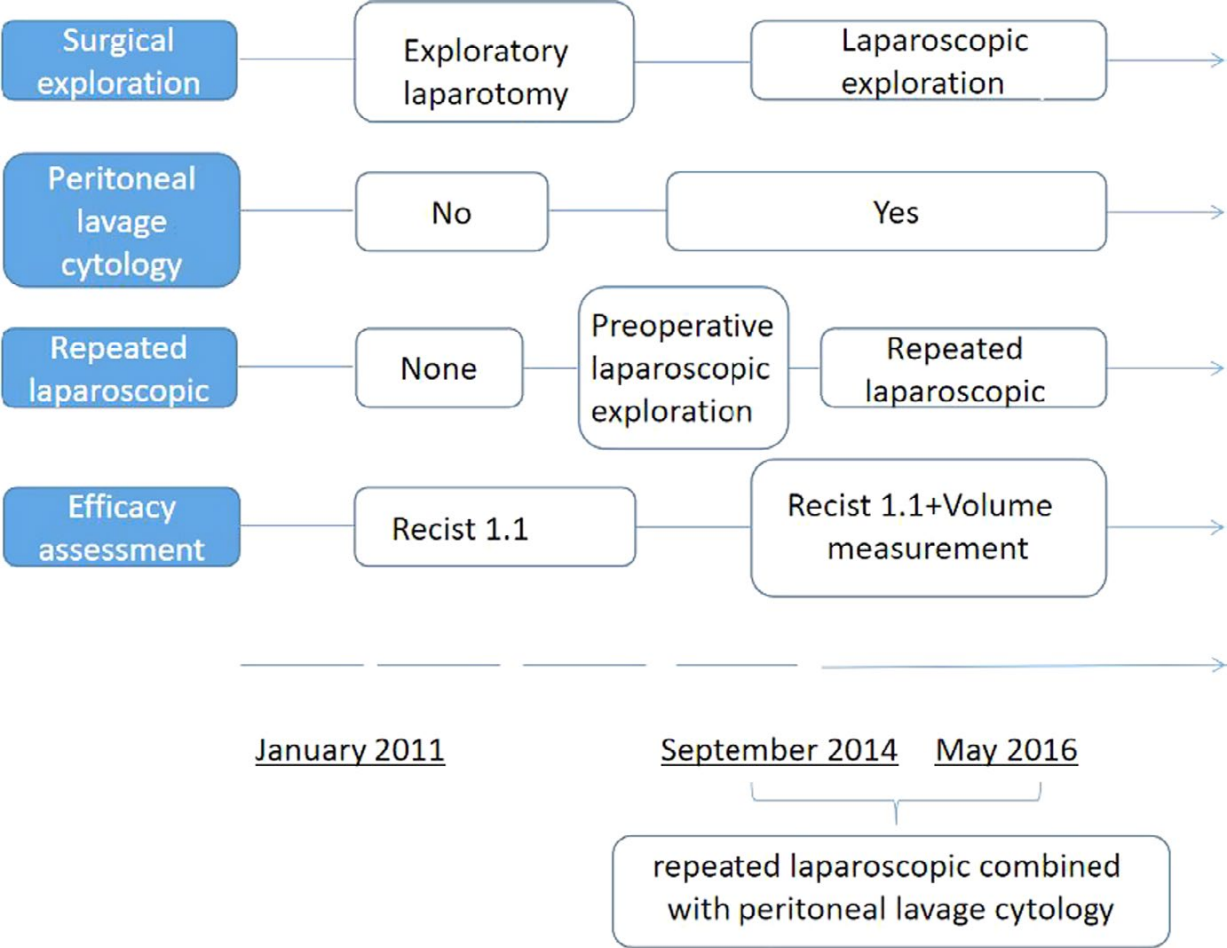
The transition and time course of surgical exploration, peritoneal lavage cytology, and efficacy assessment

**FIGURE 3:**
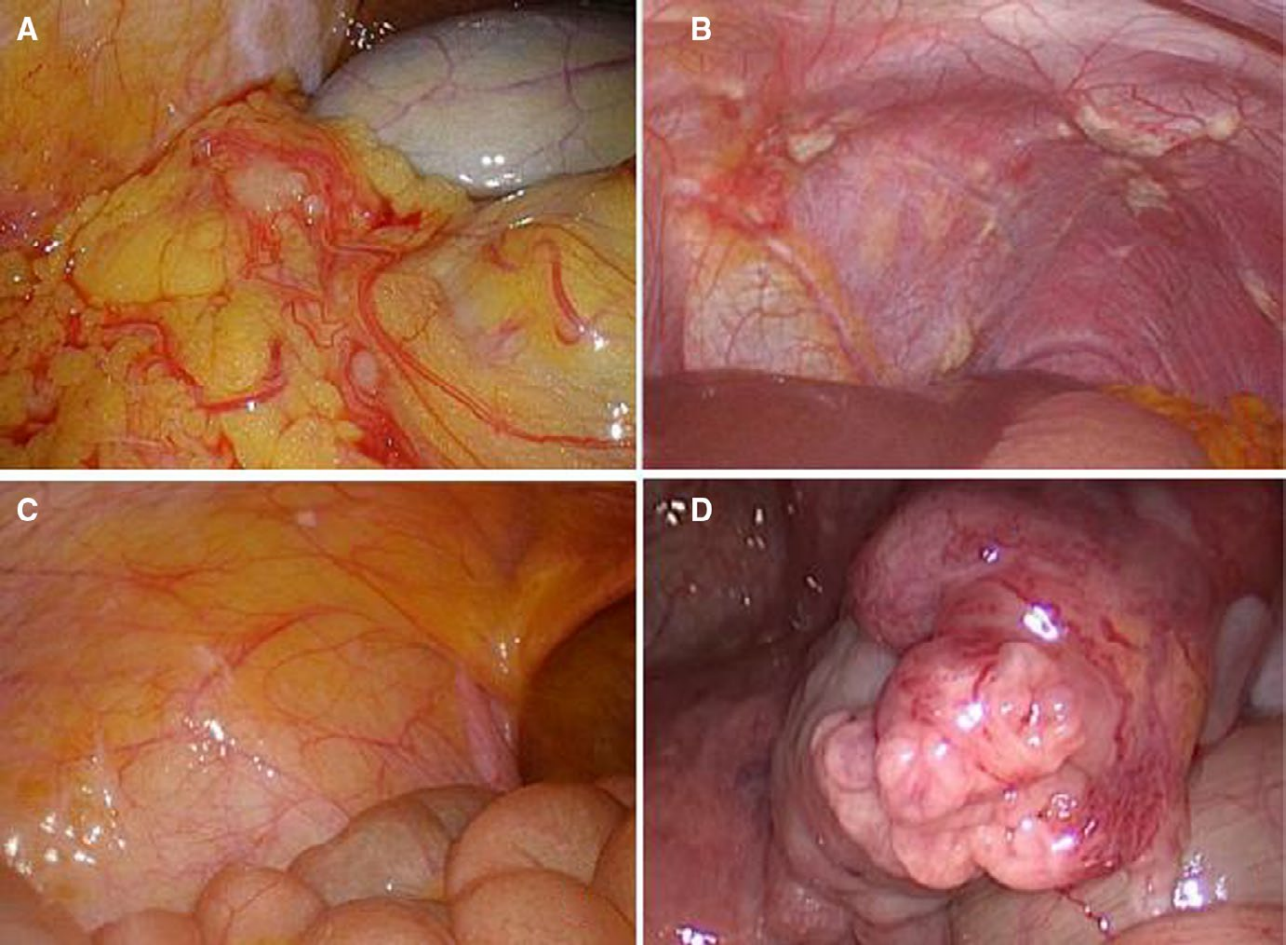
Laparoscopic exploration revealed the presence of peritoneal metastasis

**FIGURE 4:**
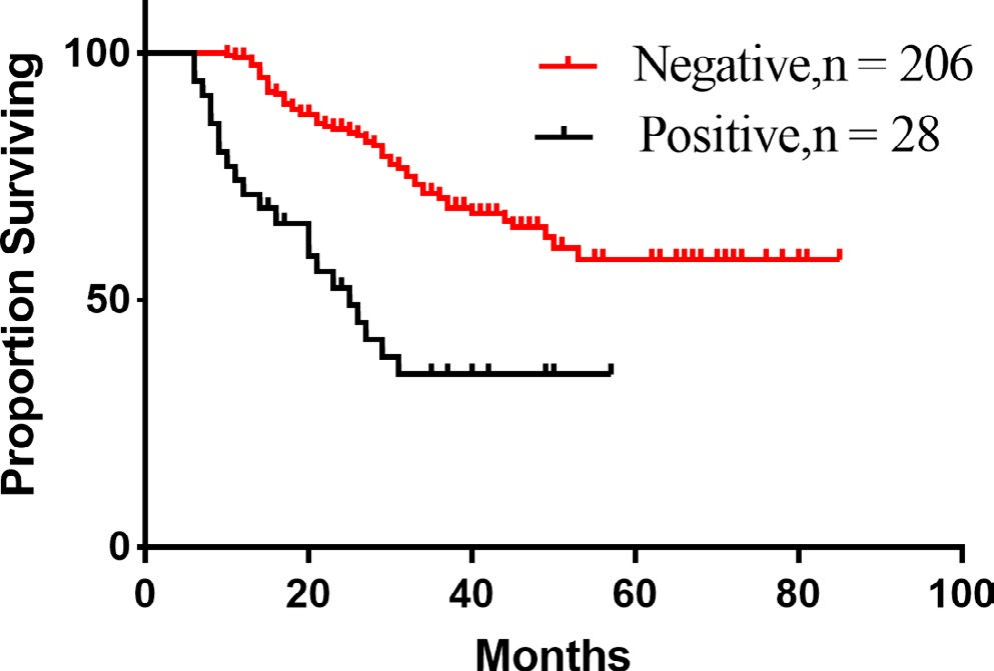
Comparison of positive and negative survival rates of peritoneal lavage cytology

Beginning in May 2014, we gradually improved the surgical exploration method, from the previous laparotomy to laparoscopic exploration, and then in September 2015, all the newly diagnosed patients were randomly enrolled before and before the operation, repeated laparoscopic exploration and staging combined with the abdominal cavity. Exfoliative cytology was performed and randomized to patients who met the enrollment criteria after screening (Figure 2). Two hundred and forty‐six patients (100 in group A, 71 in group B, and 75 in group C) underwent repeated laparoscopic exploration combined with abdominal exfoliative cytology, in 17 (12.0%), 13 (14.08%), and 15 patients, respectively. (Figure 3). Occult M1 disease was found in patients (14.67%). Among occult M1 patients, metastatic disease included peritoneal implantation (6 patients), omental implantation (5 patients) and liver invasion (3 patients), ovarian implant metastasis (3 patients), and 28 (12.33%) patients found a positive peritoneal cytology test (CY1) results. Exclusion of 17 macroscopic M1 patients, survival analysis of patients with and without CY1, found that the prognosis of patients without CY1 was significantly better than patients with CY1 (Figure 4, *P*_log‐rank_ < 0.05).

There are two cases of simple CY1: In the first case, CY1 found in the initial diagnosis of laparoscopic exploration, a total of 12 patients were not included in the group; in the second case, CY1 found in repeat laparoscopic exploration after neoadjuvant therapy, a total of 16 for this reason, the patient was judged to have progressed after treatment and the group was excluded for follow‐up treatment.

### 3.2 The clinical efficacy of SOX and XELOX as NAC

The 223 patients in group B had a total of 522 cycles of preoperative chemotherapy (mean 2.34 cycles). Group C had a total of 568 cycles of preoperative chemotherapy (mean 2.41 cycles). The tumor response in group B included CR 4.5% (10/223), PR 45.3% (101/223), SD 40.8% (91/223), PD 9.4% (21/223), ORR 49.8% (111/223), and DCR 90.5% (202/223). The tumor response in group C included CR 1.7% (4/236), PR46.2% (109/236), SD 40.3% (95/236), PD 11.9% (28/236), ORR 47.9% (113/236), DCR 88.1% (208/236). The total efficacy of group B and group C was 49.8% and 47.9%, respectively, with no statistical difference (*P* = .685). The DCRs were 90.5% and 88.1% of group B and group C, respectively, with no statistical difference (*P* = .396) (Table 4).

**TABLE 4:** Comparison of the short‐term efficacy of neoadjuvant chemotherapy between the two groups

Groups	n	CR	PR	SD	PD	ORR	DCR
B	223	10	101	91	21	111	202
C	236	4	109	95	28	113	208
*χ* ^2^		3.017	0.037	0.015	0.720	0.165	0.720
*P*		.082	.847	.904	.396	.685	.396

Abbreviations: CR, complete response; DCR, disease control rate; ORR, overall response rate; PD, progressive disease; PR, partial response; SD, stable disease.

**FIGURE 5:**
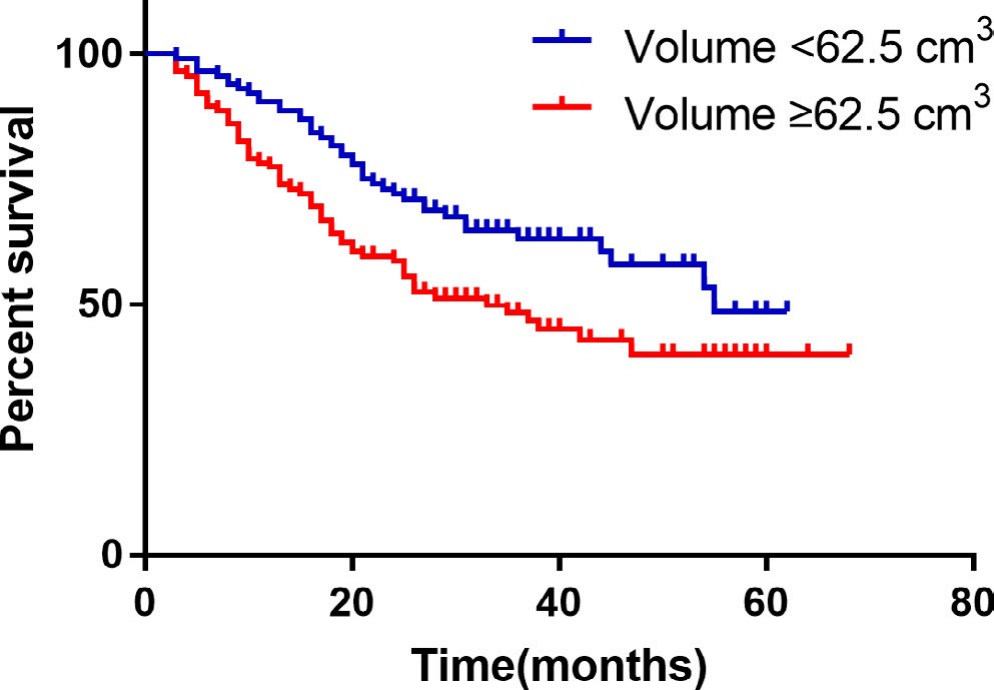
The IIIC staging patients were stratified according to the median volume of 62.5 cm^3^, and the overall survival of the two groups of patients was compared

Tumor volume measurement was used as an imaging evaluation method for clinical staging and efficacy evaluation. The tumors of 86 patients with T4a in group A were measured by volume, and IIIC patients were stratified according to the median volume of 62.5 cm^3^, and the survival curve and tumor volume were drawn. Patients with large size tumor have a significantly worse survival than those with small size (Figure 5; *P* = .009). The 116 new assisted patients in group C were evaluated by Recist standard and volume measurement, and Spearman test was used to analyze the correlation between pathology and pathological regression. The latter and pathological regression criteria were found. The correlation is stronger (*R* = .547 > .36). According to the

**FIGURE 6:**
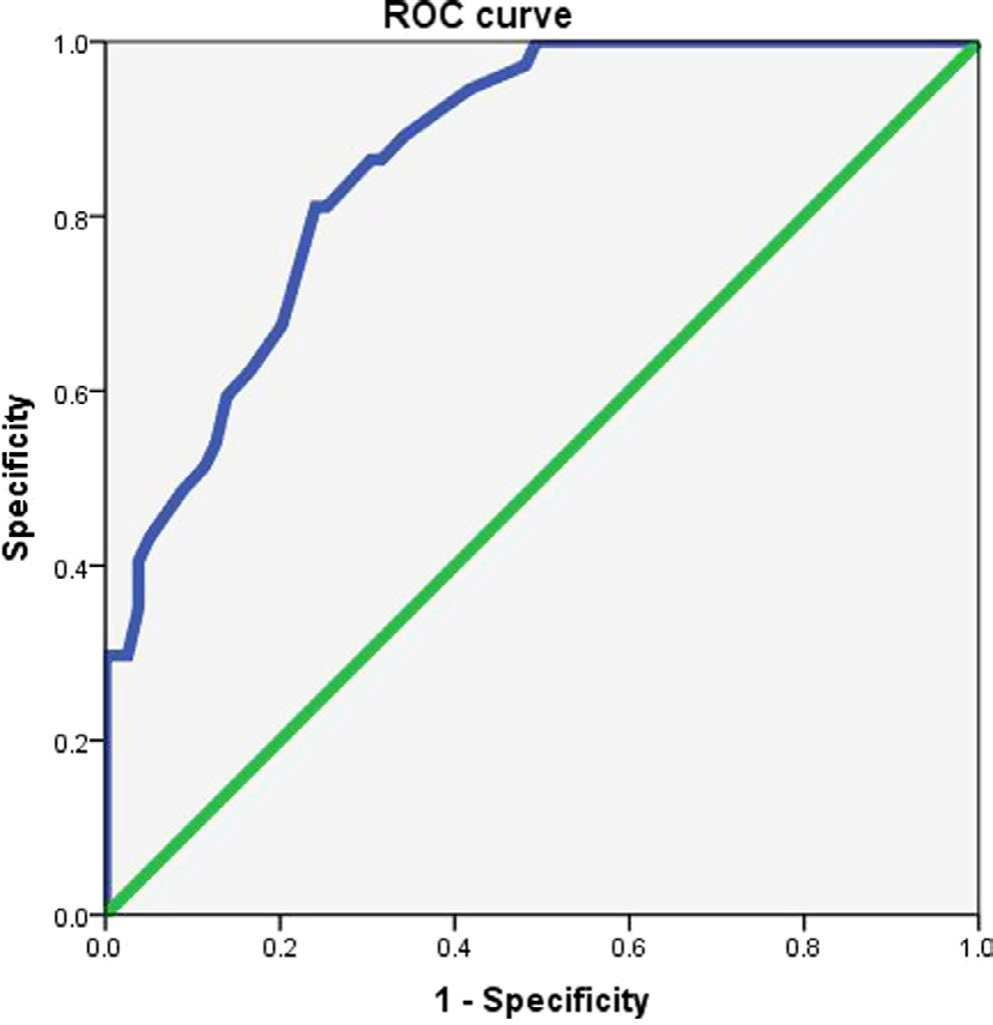
Evaluation of the percentage change of primary gastric cancer volume in ROC curve analysis for the effectiveness of chemotherapy

**FIGURE 7:**
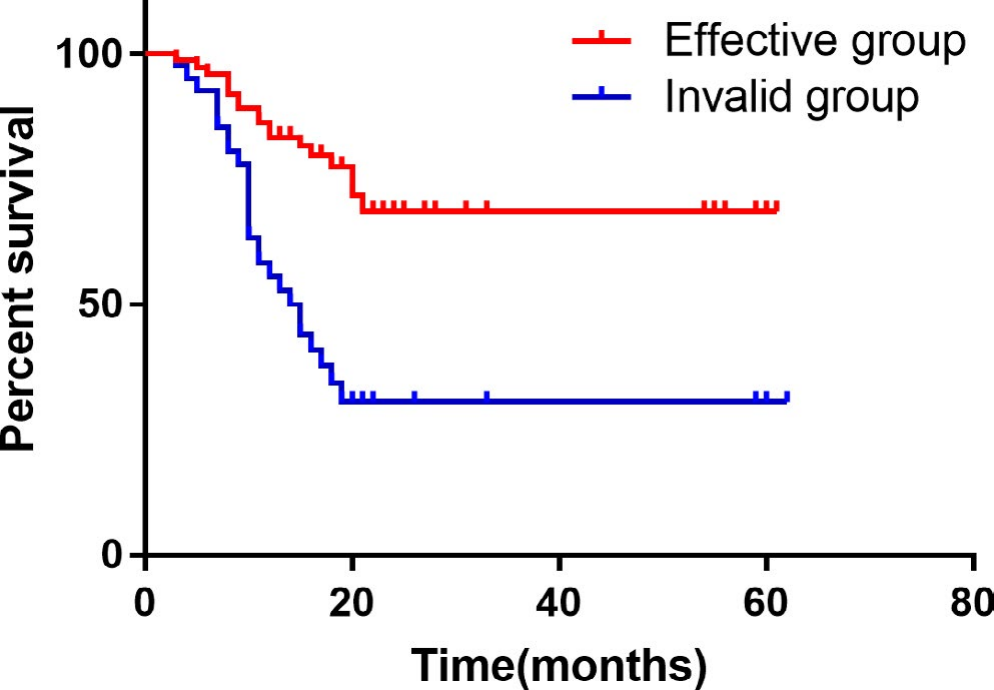
Comparison of postoperative survival rate between chemotherapy‐effective group and chemotherapy‐ineffective group in the percentage change of volume change

Receiver operating characteristic（ROC） curve analysis, the tumor volume reduction rate of 12.5% was used as the effective threshold for evaluating NAC. The chemotherapy patients were divided into effective group and ineffective group. At this time, the sensitivity and specificity of evaluation efficiency were the best, 81.1%, 75.9%, respectively (Figure 6). Survival curves were also generated, and the OS of the chemotherapy‐effective group was significantly longer than that of the ineffective group (*P*_log‐rank_ < .05). According to the traditional Recist1.1 standard, the median survival time of the effective group and the ineffective group were 25 and 20 months, respectively. The 2‐year survival rate was 65.3% and 66.7%, respectively. There was no significant difference between the two groups (*P* > .05; Figure 7). According to the new grading criteria, the median survival time of the effective and ineffective groups was 25 and 18 months, respectively, and the 2‐year survival rates were 73.3% and 51.2%, respectively. The difference between the two groups was statistically significant (*P* < .05).

## 4 Comparison of AEs between the A, B, and C groups

### 4.1 Comparison of AEs between the A and B groups

The incidence of hematologic AEs in group B including I‐II degree of thrombocytopenia and III‐IV degree of increased creatinine was higher than that in group A with statistical difference (*P*＜0.05) (Table 5A).

**TABLE 5A:** Incidence of postoperative adjuvant chemotherapy adverse events group A and group B (*x* ± *s*)

	Group A (%) N = 249		Group B (%) N = 223		*χ 2*		*P*	
	1～2	3～4	1～2	3～4	1～2	3～4	1～2	3～4
Hematological								
Leukopenia	98 (40.33)	7 (2.88)	95 (42.60)	10 (4.48)	0.247	0.851	.619	.356
Neutropenia	68 (27.98)	35 (14.40)	59 (26.46)	31 (13.90)	0.137	0.024	.712	.877
Anemia	121 (49.79)	29 (11.93)	109 (48.88)	18 (8.07)	0.039	1.913	.843	.167
Thrombocytopenia	76 (31.28)	5 (2.06)	101 (45.29)	5 (2.24)	9.697	0.021	.002	.886
AST or ALT abnormality	119 (48.97)	24 (9.88)	123 (55.16)	18 (8.07)	1.903	0.462	.168	.497
Total bilirubin increase	98 (40.33)	13 (5.35)	103 (46.19)	17 (7.62)	1.627	1.021	.202	.312
Creatinine elevation	126 (51.85)	3 (1.23)	108 (48.43)	10 (4.48)	0.544	4.528	.461	.033
Nonhematological								
Nausea	128 (52.67)	10 (4.11)	106 (47.53)	10 (4.48)	1.230	0.039	.268	.844
Vomiting	78 (32.10)	9 (0)	73 (32.74)	10 (4.48)	0.022	0.181	.883	.670
Constipation	38 (15.64)	5 (3.70)	66 (29.60)	10 (4.48)	13.07	2.198	<.001	.138
Hand‐foot syndrome	104 (42.80)	14 (5.76)	91 (40.80)	17 (7.62)	0.189	0.649	.663	.420

### 4.2 Comparison of AEs between the B and C groups

The incidence of hematologic AEs including I‐II degree of leukopenia, increased total hemoglobin levels, and III‐IV degree of neutropenia in the SOX group was higher than that in the XELOX group with statistical difference (*P* ＜ .05). The occurrence of nausea and hand‐foot syndrome in the XELOX group was higher than that in the SOX group (Table 5b), while the incidence of postoperative constipation in the SOX group was higher than that in the XELOX group.

**TABLE 5B:** Incidence of postoperative adjuvant chemotherapy adverse events in group B and group C ( $\bar{x}$ ± *s*)

	Group B (%) N = 223		Group C (%) N = 236		*χ 2*		*P*	
	1～2	3～4	1～2	3～4	1～2	3～4	1～2	3～4
Hematological								
Leukopenia	95 (42.60)	10 (4.48)	68 (28.81)	7 (2.96)	9.517	0.741	.002	.389
Neutropenia	59 (26.46)	31 (13.90)	54 (22.88)	14 (5.93)	0.790	8.235	.374	.004
Anemia	109 (48.88)	18 (8.07)	134 (56.77)	29 (12.29)	2.709	2.218	.100	.136
Thrombocytopenia	101 (45.29)	5 (2.24)	93 (39.40)	8 (3.39)	1.627	0.549	.202	.459
AST or ALT abnormality	123 (55.16)	18 (8.07)	105 (44.49)	10 (4.24)	5.217	2.943	.014	.086
Total bilirubin increase	103 (46.19)	17 (7.62)	76 (32.20)	12 (5.08)	9.426	1.248	.002	.264
Creatinine elevation	108 (48.43)	10 (4.48)	98 (41.52)	11 (4.66)	2.210	0.008	.137	.928
Nonhematological								
Nausea	106 (47.53)	10 (4.48)	127 (53.81)	30 (12.71)	2.217	9.752	.136	.002
Vomiting	73 (32.74)	10 (4.48)	62 (26.27)	20 (8.47)	2.308	2.989	.129	.084
Constipation	66 (29.60)	10 (4.48)	35 (14.83)	7 (2.97)	14.566	0.741	<.001	.389
Hand‐foot syndrome	91 (40.80)	17 (7.62)	98 (41.53)	46 (19.49)	0.024	13.638	.876	<.001

### 4.3 Comparison of preoperative and postoperative chemotherapy adverse reactions in groups B and C

We also compared the adverse reactions between preoperative and postoperative chemotherapy in groups B and C. The postoperative III‐IV degree of neutrophil count, elevated liver enzymes, elevated total bilirubin, and creatinine in group B. The incidence of elevation and hand‐foot syndrome was higher than that before surgery, and the difference was statistically significant (*P* < .05). Comparison of preoperative and postoperative chemotherapy adverse reactions in group C: postoperative III‐IV neutropenia reduction, liver enzymes, elevated total bilirubin, elevated creatinine, and incidence of nausea and hand‐foot syndrome were higher than preoperative the difference was statistically significant (*P* < .05).

### 4.4 Follow‐ups and survival analysis

Follow‐ups ceased after the death of patient or July 1, 2017. The data about the patients who survived after July 1, 2017 were censored. For those who lost to follow‐up, the last follow‐up date was treated as censored data. The medium is 20.6 months (IQR 12.0‐29.4).

**FIGURE 8:**
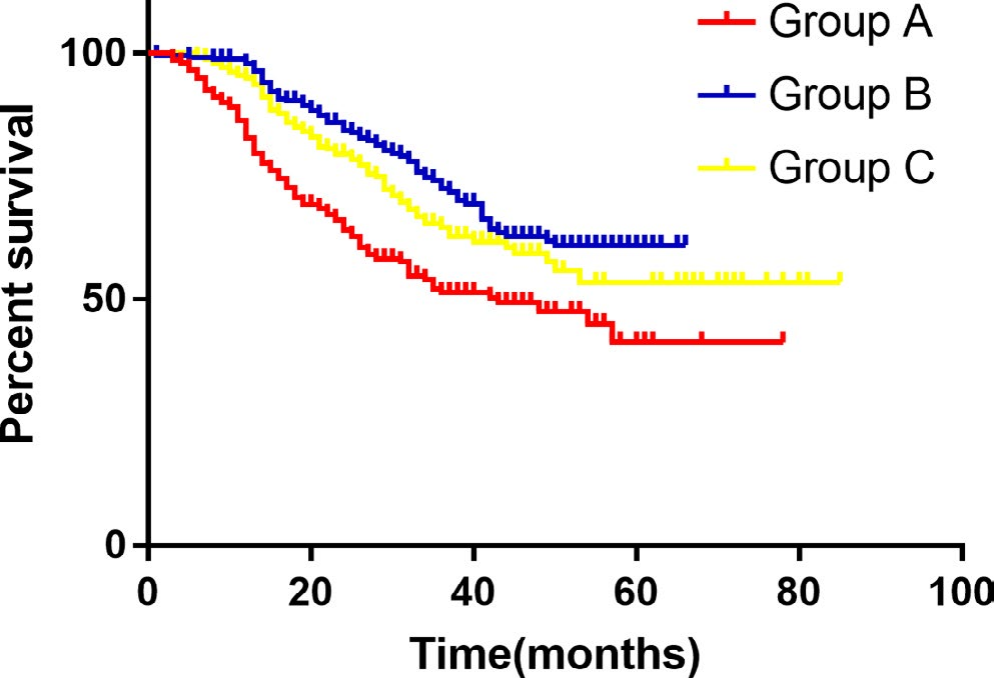
Comparison of overall survival between the three groups

A total of 225 patients were recruited in group A with the medium OS of 25 months (2‐66 months). The survival rates are 84.4% 1 year after surgery and 70.0% 2 years post operation, respectively, in Groups A. While there are 201 patients in group B with a medium OS of 29.5 months (5‐64 months) and the survival rates are 95.7% at 1 year and 86.7% at 2 years, respectively. In group C, there are 198 patients were included with a medium OS of 26.5 months (3‐80 months) and a survival rate of 92.1% at 1 year and 80.6% at 2‐year. A significant difference was found between group A and group B (*P* = .000) with no difference between group B and group C (*P* = .189), (Figure 8).

**FIGURE 9:**
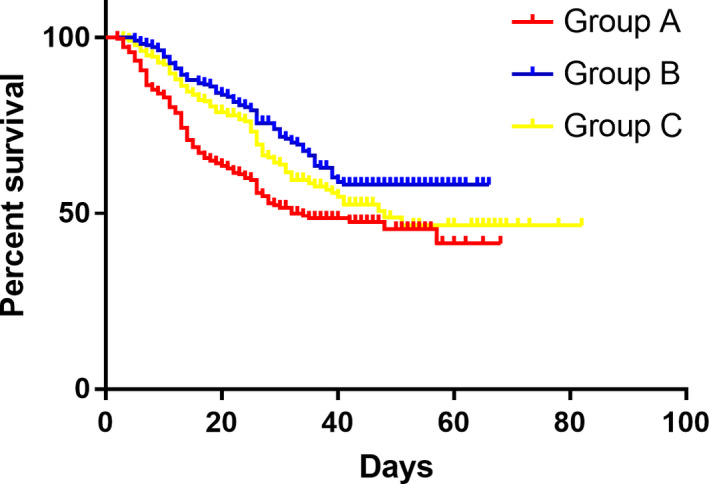
Comparison of disease‐free survival between the three groups

The median DFS in group A was 25.0 month (1‐66 months) with 1‐year disease‐free rate being 79.8% and 2‐year disease‐free rate being 66.6% while the median DFS in Group B was 28.0 month (range, from 5‐64 months) and the disease‐free rate was 92.3% at 1 year and 82.4% at 2 year, respectively. In group B the median DFS was 26.0 (range 3‐80 months) with a 1‐year disease‐free rate being 90.8% and 2‐year disease‐free being 80.0%, respectively. A significant difference was found between group A and group B (*P* = .000) with nonsignificant difference between group B and group C (*P* = .196), (Figure 9). Hazard Ratio was compared between and 0.72 of DFS was obtained (95% CI, 0.67‐0.77, *P*_noninferiority_ <.0001, *P*_log‐rank_ = .064). Besides, the upper threshold was lower than predefined of 1.38. Therefore, our data showed that noninferiority of SOX relative to XELOX.

### 4.5 Subgroup analysis

#### 4.5.1 Clinical analysis of groups B and C

Further stratified analysis of patients in groups B and C, calculating the risk of recurrence and 95% confidence interval in each subgroup, using stata 15.0 to map the forest, showing two groups of patients in IIA, IIB and IIIA, IIIB. There was no significant difference in risk of recurrence (*P* > .05). However, among patients in stage IIIC, the risk of recurrence was significantly lower in group B than in group C. The recurrence risk HRs were 1.935 (95% CI: 0.967‐3.869; *P* = .048).

#### 4.5.2 Layered analysis of Lauren classification in groups B and C

**FIGURE 10:**
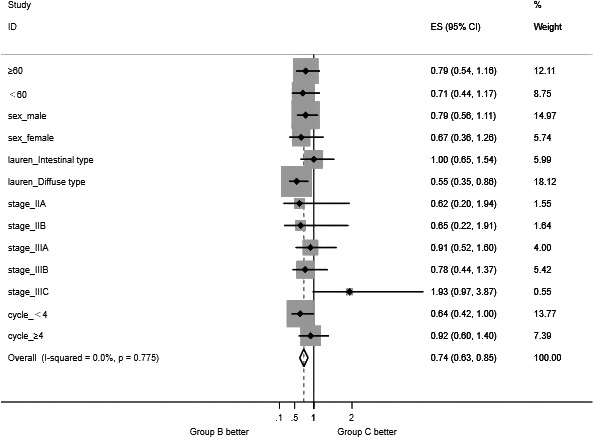
Forest plot graph of subgroup analysis (group B vs group C)

Different Lauren classifications were performed in the subgroup analysis. Among the Lauren diffuse patients, the risk of recurrence of SOX NAC was significantly lower than that of the XELOX group (*P* < .05). The recurrence risk values of the two groups were 0.55 (95% CI: 0.352‐0.860; *P* = .006), and there was no statistically significant difference in the risk of recurrence between Lauren's bowel and mixed patients (Figure 10).

## 5 DISCUSSION

The Real‐2 clinical trial from the UK further demonstrates that CAP can replace 5‐FU in the treatment of GC and L‐OHP can replace PDD in therapy. ACTS‐GC study,^4^ Korea's CLASSIC study,^5^ and INT‐0116 ^9^ study have provided sound evidence to conform that adjacent chemotherapy can improve survival rate for patients with advanced GC (stage II and III) significantly. Compared to traditional chemotherapy postoperatively, preoperative NAC can result in downstaging, reduced intraoperative dissemination, and enhanced R0 resection rates, which all improve the prognosis. Another important role of NAC is to evaluate the effect of the NAC regimen to guide the selection of the postoperative chemotherapy approach,^10^, ^11^ and following studies such as ARTIST and EORTC 40 954 has found no significant survival benefits for AGC, while EORTC 40954 demonstrated an increase in the radical resection rate in favor of T3‐4N + M0 AGC undergoing NAC.^12^, ^13^ Koizumiret et al^14^, ^15^, ^16^, ^17^ have found that the effective rate of SOX was 53%‐59% in their clinic trails. A recent phase II clinical study^18^ in Japan showed that nacG‐SOX130 has demonstrated favorable clinical safety, as it resulted in high clinical and pathological responses without compromising surgical treatment in Stage III GC. Clinical RR was 88.2% (95% CI: 76.8‐99.6) with CRs of (8.8%). While in this study, the effective rates were 49.8% and 47.9% in group B and group C, respectively, which was consistent with Koizumiret's study. The degrade rates were 55.6% and 50.4% in group B and group C, respectively. The R0 removal rates were 81.7%, 88.7%, and 83.1% in group A, group B, and group C, respectively. The results showed that there was statistical difference between group A and group B (*P* < .05), whereas no difference was found between group A and group B (*P* > .05). In this study, we discovered that NAC, both SOX and XELOX regimen, can result in downstaging and enhanced R0 resection rate in terms of short‐term effect. Although there was no statistical difference in the R0 surgical resection rate between the preoperative XELOX group and the direct surgery group, there was still a trend to improve the degree of radical cure. The above results indicate that the results of this study are consistent with previous studies. For long‐term effect, the average survival time was 25.0 months in group A and 29.5 months in group B, respectively, while the DFS was 25.0 and 28 months in group A and group B, respectively. According to these results, we concluded that using SOX perioperatively can improve both median survival time and median DFS time compared to traditional SOX method (only administered postoperatively). There was no statistical difference between the protocol and the total number of medication cycles, indicating that preoperative neoadjuvant therapy combined with surgery and PAC can improve the long‐term efficacy of patients compared with direct surgery plus PAC.

With the development of laparoscopic technology, staged laparoscopic exploration was adapted to replace the original exploratory laparotomy, while repeated laparoscopic exploration were performed on patients with NAC. Yoshida^19^ suggested that staging laparoscopy for patients with advanced gastric cancer, while stage IV GC could be divided into four categories according to the presence or absence of peritoneal metastasis, which could not directly benefit from surgery. As a supplement to computed tomography（CT）, magnetic resonance imaging（MRI）, and positron emission tomography‐computed tomography（ PET‐CT）, staged laparoscopic exploration could be more accurate in clinical staging. Through laparoscopic exploration, unnecessary exploratory laparotomy could be avoided, and surgically related complications can be reduced, so that truly individualized treatment could be achieved. Repetitive laparoscopic exploration^20^ has increasingly become a supplement to clinical efficacy evaluation.

There is no standardized treatment plan for advanced gastric cancer at present, and some studies suggest that XELOX is a first‐line chemotherapy for GC. There are also studies suggesting that S‐1 treatment is better than Xeloda and has fewer side effects. However, different pathological subtypes are associated with the prognosis of GC and the choice of chemotherapy drugs. There is currently no evidence that teguin is superior to Xeloda or intravenous 5‐FU in patients with intestinal type GC. The number of studies investigating drug selection based on different pathological types is currently limited, and most of these studies are observational studies, phase II clinical trials or retrospective analyses. In the Japanese Clinical Oncology Group 9912 trial,^21^ no significant difference in median survival time was found between 5‐FU, CAP, and S‐1 regimens. However, subgroup analyses indicate that S‐1 and CAP are more effective than 5‐FU in the treatment of diffuse GC. Ichikawa et al^22^ found that diffuse dihydropyrimidine dehydrogenase (DPD) mRNA levels were significantly higher than intestinal type. This may be due to the fact that the S‐1 component contains DPD enzyme to inhibit gemcitabine, so patients with diffuse GC are better than CAP or 5‐FU. S‐1 is a third‐generation fluorouracil derivative anticancer drug consisting of tegafur, gemcitabine, and ethaceta potassium at 1/0.4/1 (molar ratio), and tegafur is 5‐FU prodrug is slowly converted to 5‐FU by the action of cytochrome P450 in the liver to exert an antitumor effect. Gemex is mainly distributed in the liver, 5‐FU catabolic enzyme DPD, which selectively antagonizes gemcitabine, thereby reducing the inactivation of 5‐FU, and then making the concentration of 5‐FU‐phosphorylated metabolite (5‐FUMP) is increased, which enhances the antitumor effect. Otticept potassium can specifically inhibit the orotidine ribosyltransferase in intestinal mucosa and block the phosphorylation of 5‐FU. The phosphorylation product of 5‐FU can cause 5‐FU gastrointestinal adverse reactions. In this study, subgroup analysis was performed on different Lauren classifications in patients in groups B and C. According to the risk of recurrence, the forest map showed that patients with Lauren diffuse type had significantly lower risk of recurrence than those in XELOX group (*P* < .05).

In order to further investigate the safety of adjuvant chemotherapy, we investigated and compared the adverse effects and chemotherapy adherence rate in group A, group B, and group C. Since we reviewed that patients received adjacent chemotherapy had less adverse effects with low severity, the adjacent chemotherapy was safe and facilitated the chemotherapy compliance perioperatively. In this study, preoperative and postoperative adverse reactions of chemotherapy in group B and group C were being compared, which showed that the postoperative incidence of both hematological adverse reactions and nonhematological adverse reactions were higher than preoperative, which was possibly caused by the reconstruction of digestive tract, the reduction of gastric volume, and a long period of fasting after gastrectomy. Therefore, malnutrition not only weakens the efficacy of chemotherapy, but also increases the risk of adverse drug reactions. We also discovered that a higher incidence of the III‐IV hand‐foot syndrome (a nonhemolytic side effect) occurred in patients who accepted XELOX than those received SOX in group C. This is because the hand‐foot syndrome is the common side effect of Antimetabolite chemotherapy agents, such as CAP, cyclophosphamide, vinorelbine. Among these agents, CAP is mostly like to cause the hand‐foot syndrome.^23^ Though a comparison between group B and group C, the results showed the rate of side effects was higher in the patients received SOX than those treated with XELOX and a lower adherence rate was also detected in patients treated with SOX. Analysis of adherence rate may cause by the high dosage of S1 in SOX regiment as this can lead to a high rate of side effects resulting in poor adherence rate. Therefore, the dosage of S1 needs to be adjusted in order to improve adherence later on.

This study showed that both survival time and disease‐free time have extended in groups received C‐S‐C model adjuvant chemotherapy. The tendency that the AGC patients treated with both SOX and XELOX of C‐S‐C model had an extended DFS and OS was observed in this study (a separation was found in Kaplan‐Meier curve). That result is consistent with the previous clinic trail results.

Due to the limited study time, some patients were not followed up more than 3 years and the number of recurrent cases was also limited. Therefore, further researches are needed to deeply investigate the effects of SOX and XELOX on AGC patents’ recurrence and prognosis. We participate more results will be published via multicentered prospectively randomized controlled stage III clinic trail, so more evidence will be provided to assist patients to reach optimal treatment outcome.

## CONFLICTS OF INTEREST

The authors have no conflicts of interest to declare.

## AUTHOR CONTRIBUTIONS

Qun Zhao, Yong Li contributed to the study design. Qun Zhao, Changhong Lian, Zhibin Huo, Ming Li, Yang Liu contributed to the data collection and analysis. Qun Zhao and Yang Liu led the analysis and interpretation. Qun Zhao, Yang Liu and Yong Li contributed to the writing of the paper. All authors contributed to review of paper and approved the final version.

## ETHICAL STATEMENT

The authors are accountable for all aspects of the work in ensuring that questions related to the accuracy or integrity of any part of the work are appropriately investigated and resolved.

## Supporting information

Supplementary Material

## APPENDIX 1

### PATIENTS RECRUITMENT CRITERIA

#### Inclusive criteria

1. above the age of 18;
2. pathology conformed the Gastric cancer diagnosis (include carcinoma of gastric cardia and fundus)
3. participants did not receive anticancer therapy including chemotherapy and radiotherapy previously
4. behavior: ECOG ≦2;
5. gastric cancer staging: Stage III、Stage IV;
6. no distant organ metastasis (M0);
7. consent signed up

#### Exclusive criteria

1. patients who are allergic to S1, capecitabine, oxaliplatin;
2. patients with severe bone marrow suppression: (a) white cells less than 4000/mm^3^; (b) neutrophils less than 2000/mm^3^; and (c) platelet less than 100 000/mm^3^;
3. patients with severe renal impairment or hepatic dysfunction: (a) bilirubin value is two times greater than the normal upper limits; (b) ALT and AST in patients without liver metastasis is 2.5 times greater and in patients suffering from live metastasis is five times greater than the normal upper limit; and (c) the value of serum creatinine is two times greater than the normal upper limit;
4. patients with peripheral neural disease;
5. patients who are receiving other antineoplastic agents such as fluorouracil (including the combination drugs);
6. female patients in pregnancy or lactation or patients in child‐bearing age unwilling to take contraceptive measures (including males);
7. patients with unstable mental issues which may affect their understanding of the chemotherapy in this study;
8. patients had Myocardic infarction, or existing severe/ unstable angina, hear function insufficiency within 6 months;
9. severe disorders of organs such as severe COPD, Interstitial pneumonia, pulmonary fibrosis or combining respiratory failure, kidney function insufficiency or glomerulosclerosis unmanageable diabetes;
10. severe infection needs hospitalization;
11. patients with dysphasia, active digestive ulcer, digestive track bleeding, and perforation.

#### Drop criteria

1. the deterioration of patient's condition or recurrence occurs;
2. patients withdraw the concert;
3. the doctor in charge considers the treatment needs to cease duo to severe drug side effects;
4. the doctor in charge considers the treatment needs to cease duo to severe complications;
5. the doctor in charge considers ceasing treatment is the best option in behalf of the patient.

## References

[1] International Agency for Research on Cancer (IARC) . GLOBOCAN 2018: estimated cancer incidence, mortality and prevalence worldwide in 2018; 2018.

[2] Yamashita K , Kurokawa Y , Yamamoto K , et al. Risk factors for poor compliance with adjuvant S‐1 chemotherapy for gastric cancer: a multicenter retrospective study. Ann Surg Oncol. 2017;24:2639‐2645. 28608116 10.1245/s10434-017-5923-2

[3] Bang Y‐J , Kim Y‐W , Yang H‐K , et al. Adjuvant capecitabine and oxaliplatin for gastric cancer after D2 gastrectomy (CLASSIC): a phase 3 open‐label, randomised controlled trial. Lancet. 2012;379:315‐321. 22226517 10.1016/S0140-6736(11)61873-4

[4] Sasako M , Sakuramoto S , Katai H , et al. Five‐year outcomes of a randomized phase III trial comparing adjuvant chemotherapy with S‐1 versus surgery alone in stage II or III gastric cancer. J Clin Oncol. 2011;29:4387‐4393. 22010012 10.1200/JCO.2011.36.5908

[5] Cunningham D , Allum WH , Stenning SP , et al. Perioperative chemotherapy versus surgery alone for resectable gastroesophageal cancer. N Engl J Med. 2006;355:11‐20. 16822992 10.1056/NEJMoa055531

[6] Zhao Q , Li Y , Tian Y , et al. Histological complete response after neoadjuvant XELOX in advanced gastric carcinoma. Hepatogastroenterology. 2013;60:638‐640. 23340232 10.5754/hge121131

[7] Zhao Q , Li Y , Huang J , et al. Short‐term curative effect of S‐1 plus oxaliplatin as perioperative chemotherapy for locally advanced gastric cancer: a prospective comparison study. Pharmazie. 2017;72:236‐240. 29441995 10.1691/ph.2017.6865

[8] Yonemura Y , Ishibashi H , Hirano M , et al. Effects of neoadjuvant laparoscopic hyperthermic intraperitoneal chemotherapy and neoadjuvant intraperitoneal/systemic chemotherapy on peritoneal metastases from gastric cancer. Ann Surg Oncol. 2017;24:478‐485. 27506661 10.1245/s10434-016-5487-6

[9] Macdonald JS , Smalley SR , Benedetti J , et al. Chemoradiotherapy after surgery compared with surgery alone for adenocarcinoma of the stomach or gastroesophageal junction. N Engl J Med. 2001;345:725‐730. 11547741 10.1056/NEJMoa010187

[10] Aoyama T , Nishikawa K , Fujitani K , et al. Early results of a randomized two‐by‐two factorial phase II trial comparing neoadjuvant chemotherapy with two and four courses of cisplatin/S‐1 and docetaxel/cisplatin/S‐1 as neoadjuvant chemotherapy for locally advanced gastric cancer. Ann Oncol. 2017;28:1876‐1881. 28486692 10.1093/annonc/mdx236

[11] Palmela C , Velho S , Agostinho L , et al. Body composition as a prognostic factor of neoadjuvant chemotherapy toxicity and outcome in patients with locally advanced gastric cancer. J Gastric Cancer. 2017;17:74‐87. 28337365 10.5230/jgc.2017.17.e8 PMC5362836

[12] Ychou M , Boige V , Pignon J‐P , et al. Perioperative chemotherapy compared with surgery alone for resectable gastroesophageal adenocarcinoma: an FNCLCC and FFCD multicenter phase III trial. J Clin Oncol. 2011;29:1715‐1721. 21444866 10.1200/JCO.2010.33.0597

[13] Xu W , Beeharry MK , Liu WT , Yan M , Zhu ZG . Preoperative chemotherapy for gastric cancer: personal interventions and precision medicine. Biomed Res Int. 2016;2016:3923585. 28105420 10.1155/2016/3923585 PMC5220419

[14] Schuhmacher C , Gretschel S , Lordick F , et al. Neoadjuvant chemotherapy compared with surgery alone for locally advanced cancer of the stomach and cardia: European Organisation for Research and Treatment of Cancer randomized trial 40954. J Clin Oncol. 2010;28:5210‐5218. 21060024 10.1200/JCO.2009.26.6114 PMC3020693

[15] Koizumi W , Narahara H , Hara T , et al. S‐1 plus cisplatin versus S‐1 alone for first‐line treatment of advanced gastriccancer (SPIRITS trial): a phase Ⅲ trial. Lancet Oncol. 2008;9:215‐221. 18282805 10.1016/S1470-2045(08)70035-4

[16] Koizumi W , Takiuchi H , Yamada Y , et al. Phase II study of oxaliplatin plus S‐1 as first‐line treatment for advanced gastric cancer (G‐SOX study). Ann Oncol. 2010;21:1001‐1005. 19875759 10.1093/annonc/mdp464

[17] Park I , Lee J‐L , Ryu M‐H , et al. Phase I/II and pharmacokinetic study of S‐1 and oxaliplatin in previously untreated advanced gastric cancer. Cancer Chemother Pharmacol. 2010;65:473‐480. 19551382 10.1007/s00280-009-1052-3

[18] Konishi S , Manaka D , Ikeda Y , et al. Phase II study of neoadjuvant chemotherapy with S‐1 plus oxaliplatin at a dose of 130 mg/m^2^ (nacG‐SOX130) in clinical(c)Stage III gastric cancer. Ann Oncol. 2019;30(Suppl 4):iv27‐iv28.

[19] Yoshida K , Yamaguchi K , Okumura N , et al. Is conversion therapy possible in stage IV gastric cancer: the proposal of new biological categories of classification. Gastric Cancer. 2016;19(2):329‐338. 26643880 10.1007/s10120-015-0575-z PMC4824831

[20] Thiels CA , Ikoma N , Fournier K , et al. Repeat staging laparoscopy for gastric cancer after preoperative therapy. J Surg Oncol. 2018;118:61‐67. 29878364 10.1002/jso.25094 PMC7703853

[21] Takahari D , Boku N , Mizusawa J , et al. Determination of prognostic factors in Japanese patients with advanced gastric cancer using the data from a randomized controlled trial, Japan Clinical Oncology Group 9912. Oncologist. 2014;19:358‐366. 24668328 10.1634/theoncologist.2013-0306 PMC3983816

[22] Ichikawa W , Takahashi T , Suto K , et al. Thymidylate synthase and dihydropyrimidine dehydrogenase gene expression in relation to differentiation of gastric cancer. Int J Cancer. 2004;112:967‐973. 15316940 10.1002/ijc.20511

[23] Wang JZ , Cowley A , McLellan BN . Differentiating hand‐foot syndrome from tinea in patients receiving chemotherapy. Acta Oncol. 2016;55:1061‐1064. 27181134 10.3109/0284186X.2016.1155739

