# Supplementary material for: The efficacy and safety of neoadjuvant chemotherapy on patients with advanced gastric cancer: A multicenter randomized clinical trial
Source: Cancer Med. 2020 Jun 24;9(16):5731–45. doi: 10.1002/cam4.3224 (PMC7433829; doi:10.1002/cam4.3224)
Supplement: Supplementary file 1 — Supplementary Material [file CAM4-9-5731-s001.docx]

Protocal

Research Program Number：TOTTG030103

The efficacy and safety of neoadjuvant chemotherapy on patients with advanced gastric cancer: a multicenter randomized clinical trial

Initial version：20110907
Second edition：20111020
Third edition：20111211

Revised version

No. Date

1 20120206
2 20120220

3 20120501

Affirmation: Confidential information, please keep it confidential and cannot be used for other purposes.

**Table of Contents**

1. Research background

2. research design.

1) Research Purpose

2)Research design

3. Observation items and evaluation

1)Evaluation of efficacy

2)Safety evaluation

2.1 Evaluation of toxic and side effects

4. Aiswan dosage and adjustment

5. Statistical analysis plan

6. References

7. Accessories:

Attachment 1: Physical condition scoring criteria

Attachment 2: Evaluation Criteria for Objective Curative Effects of Cancer Drugs

Attachment 3: Commonly used toxicity classification standards

NCI Common Toxicity Evaluation Standard (1)

Attachment 4: Tumor staging standards

Attachment 5: Instructions for patients outside the hospital

Attachment 6: Center Number

1. Research background

Gastric cancer is one of the most common malignant tumors that seriously endanger human health. The number of newly diagnosed cases in the world is about 899,000 people, ranking sixth among all malignant tumors; there are about 730,000 deaths, and the death rate ranks third among all tumors. Bit (accounting for 10.3%). In China, the incidence of gastric cancer is still the second highest among all kinds of malignant tumors, with more than 460,000 newly diagnosed patients each year, accounting for nearly 47% of the world; deaths are about 350,000 per year, accounting for 18% of all malignant tumor deaths, men and women The ratio is about 2: 1; at the same time, it has been on the rise for many years, with the male mortality rate increasing by 10.98% in the 1990s and the 1970s, and the female growth rate by 6.32%.

Surgery is the preferred treatment for gastric cancer, but in most countries except Japan, early screening rates are low due to the lack of screening. In more than 50% of cases, the early symptoms are not obvious or atypical. Once diagnosed, they often show local advanced Or it may invade the peritoneum, surround the large blood vessels or distant metastasis and cannot be operated; and the tumor is limited to nearly 50% of them cannot be radically removed, so the rate of radical surgery is still low. In recent years, although the detection rate of early gastric cancer has increased, and the surgical methods and the comprehensive treatment have been actively improved and standardized, the 5-year survival rate of gastric cancer is still hovering between 20% and 30%. In addition to early gastric cancer, most gastric cancers are prone to relapse after surgery, especially those with existing lymph node metastasis. The local recurrence rate is as high as 80% or more; even early patients have recurrence and metastasis after radical resection . Therefore, gastric cancer remains a serious challenge facing the clinical oncology community.

Preoperative chemotherapy is not a new treatment method. It refers to patients with tumors who are diagnosed and then undergo two to three courses of regular chemotherapy before surgery, and rest for one to two weeks before surgery. This adjuvant chemotherapy has been used clinically for more than 20 years, but there has been no large-scale research to confirm its clinical value. Until the completion of the MAGIC trial in 2006, it was concluded that perioperative chemotherapy can increase the 5-year survival rate by 13 Percentage points.

What benefits will preoperative adjuvant chemotherapy bring to patients? Nearly 20 years of experience in preoperative adjuvant chemotherapy is believed to have advantages. Preoperative chemical drugs can make the tumor be hit and killed for the first time after the tumor occurs, so that the tumor volume can be reduced, the purpose of downgrade can be achieved, and the surgical resection rate can be improved. At the same time, it can reduce the reactive edema of the tissue and reduce the invasion and adhesion of the tumor and surrounding tissues. Preoperative chemotherapy can inhibit and kill free cancer cells that invade the bloodstream and reduce the possibility of blood metastasis. Metastasis of cancer cells to lymph nodes along the lymphatic vessels is the most common method of metastasis. The smaller the lymph node metastasis, the more difficult to find during surgery. Preoperative chemotherapy can effectively suppress small lymphatic metastases and reduce the risk of recurrence. Through preoperative adjuvant chemotherapy, understand how the tumor responds to chemotherapy drugs, to determine whether the patient needs to continue chemotherapy after surgery, and guide the patient's next treatment plan to improve the treatment effect. Reduce the clinical symptoms of some tumors in patients with advanced gastric cancer, and reduce the pain of patients before surgery. The above advantages are all based on the effectiveness of preoperative adjuvant chemotherapy. Currently, the effective rate for gastric cancer chemotherapy is only maintained at about 50%, so preoperative adjuvant chemotherapy is bound to have problems and drawbacks. For patients who do not respond to chemotherapy, disease progression may occur during preoperative adjuvant chemotherapy and treatment may be delayed. For patients with preoperative adjuvant chemotherapy, it can achieve the purpose of downgrade and improve the surgical resection rate.

In this open clinical observation program, the purpose of this study is to evaluate the safety and efficacy of esperin, capecitabine, and oxaliplatin in perioperative chemotherapy treatment for patients with advanced gastric cancer. . At present, there is no evidence at home and abroad for the efficacy and safety of pre-adjuvant neoadjuvant chemotherapy has not been proved by a large phase III clinical trial, nor is there evidence that Eswan combined with oxaliplatin and capecitabine combined with oxali Which platinum regimen is better for perioperative treatment of patients with advanced gastric cancer. In this trial, in order to explore the effectiveness and safety of perioperative chemotherapy for locally advanced gastric cancer, we will further explore the best perioperative chemotherapy for advanced gastric cancer.

2. Research Design

1. Research purpose:

1. Main objectives:

  The first stage: R0 removal rate

  Stage 2: Disease-free survival (DFS)

2. Secondary goals:

  Overall response rate (ORR), disease control rate (DCR)

  Downstaging Rate

  Overall survival (OS)

  Compliance of Aswan, Capecitabine, Oxaliplatin

  Safety

2. Research design

2.1 Research plan

This observation plan adopts a two-stage approach. Subjects who meet the entry criteria will be randomly assigned to three groups at a 1: 1: 1 ratio, and random allocation will be stratified according to tumor stage, center, and behavioral ability PS:

The first stage: neoadjuvant chemotherapy observation program.

 (Group A) No pre-adjuvant neoadjuvant chemotherapy.

 (Group B) 2 cycles of neoadjuvant chemotherapy-Eswan combined with oxaliplatin. . Aswan 40mg ~ 60mg from the first day to the 14th day of each course, twice daily after breakfast and dinner, and intravenous infusion of oxaliplatin 130 mg / m2 on the first day. The duration of treatment lasts for two treatment cycles, or continues until the subject appears to be eligible for stopping treatment.

 (Group C) Preoperative neoadjuvant chemotherapy-capecitabine combined with oxaliplatin for 2 cycles. Capecitabine 2000 mg / m2 was divided into twice daily for breakfast and dinner from day 1 to day 14 of each course After oral administration, oxaliplatin 130 mg / m2 was infused intravenously on the first day. The treatment period lasts for two treatment cycles, or continues until the subject appears to meet any conditions for stopping treatment.

Patients in the neoadjuvant chemotherapy group should undergo an objective response rate assessment after two cycles of treatment, and perform surgery within four weeks after the end of the course of treatment. At the same time, the stage of the patient's tumor needs to be reassessed. During the operation, whether the R0 resection can be completed is used as the main evaluation index in the first stage. Only patients who did not progress in neoadjuvant therapy during the first stage of observation (CR, PR, SD) entered the second stage of observation.

The second stage: Adjuvant chemotherapy observation program.

 (Group A) Postoperative adjuvant chemotherapy-Eswan combined with oxaliplatin for 8 cycles. Every day from the first day to the 14th day of each course, twice a day, each after oral and esvan 40 mg ~ 60 mg oral, and on the first day of intravenous infusion of oxaliplatin 130 mg / m2. The duration of treatment lasts for 6 treatment cycles, or continues until the subject appears to be eligible to stop treatment.

 (Group B) Postoperative adjuvant chemotherapy-Eswan combined with oxaliplatin for 6 cycles. Twice a day from the first day to the 14th day of each course of treatment, each after oral and estrogen 40mg ~ 60mg orally, and intravenous infusion of oxaliplatin 130mg / m^2^ on the first day. The duration of treatment lasts for 6 treatment cycles, or continues until the subject appears to be eligible to stop treatment.

 (Group C) Postoperative adjuvant chemotherapy-capecitabine combined with oxaliplatin for 6 cycles. Capecitabine 1000 mg / m^2^ was taken orally after breakfast and dinner twice a day from day 1 to day 14 of each treatment course, and oxaliplatin 130 mg / m^2^ was intravenously infused on the first day. The duration of treatment lasts for 6 treatment cycles, or continues until the subject appears to be eligible to stop treatment.

 Precautions for the administration of Aiswan: The actual daily dose of Aiswan will be determined according to the body surface area (BSA) of each subject, and the daily dose shall be equally divided between breakfast and dinner. However, if the administered dose cannot be divided equally, a higher dose will be administered after breakfast. Every 3 weeks is a cycle, Eswan needs to be administered a total of 28 times; Eswan is given after dinner on the first day of the course of treatment, so the administration time will continue until the 15th day after breakfast.

※Aswan standard starting dose：

| BSA | Aiswan dose (calculated according to the dose of teflurane) |
| --- | --- |
| < 1.25 mm2 | 80 mg/day |
| 1.25 mm2 To < 1.5mm2 | 100 mg/day |
| 1.5mm2 | 120 mg/day |

**Evaluation schedule**

The following table describes in detail the schedule of each inspection and evaluation of each group.

Stage 1: Neoadjuvant chemotherapyGroup A:

|  | Case | | Surgery |
| --- | --- | --- | --- |
| Detection time | -28Day | -14 Day |  |
| Oxaliplatin |  |  |  |
| Eswan |  |  |  |
| Tumor measurement | ⊙◎ |  |  |
| Laboratory examination |  | ⊙ | ⊙ |
| Electrocardiogram | ⊙ |  | ◇ |
| Chest x-ray | ⊙ |  |  |
| Subjective / objective symptoms |  | ⊙ | ⊙ |
| Vital signs |  | ▲ | ⊙ |

⊙: Necessary

●: Execute every 6 weeks after entering the case

○: Execute before or before giving Eswan or Oxaliplatin.
◇: Test when needed.

▲: Weight and physical fitness are necessary measures.

◎: Tumor measurement must be performed within 28 days before the case.

★: If the administration of esvan or oxaliplatin is interrupted due to intolerable toxicity, the subject must conduct a toxicity assessment at least once a week to determine whether the re-administration can be continued.

Group B :

|  | Case | | Neoadjuvant chemotherapy  (Repeat 2 cycles) | | | Surgery |
| --- | --- | --- | --- | --- | --- | --- |
| Detection time | -28 Day | -14 Day | Week 1 | Week 2 | Week 3 |  |
| Oxaliplatin |  |  | First Day |  |  |  |
| Eswan |  |  |  |  | Withdrawal |  |
| Tumor measurement | ⊙◎ |  |  | ●Every 6 weeks |  |  |
| Laboratory examination |  | ⊙ | ⊙ |  |  | ⊙ |
| Electrocardiogram | ⊙ |  | ◇ | ◇ | ◇ | ◇ |
| Chest x-ray | ⊙ |  |  | ●Every 6 weeks |  |  |
| Subjective / objective symptoms |  | ⊙ | ⊙ |  |  | ⊙ |
| Vital signs |  | ▲ | ⊙ |  |  | ⊙ |

⊙: Necessary

●: Execute every 6 weeks after entering the case

○: Execute before or before giving Eswan or Oxaliplatin.
◇: Test when needed.

▲: Weight and physical fitness are necessary measures.

◎: Tumor measurement must be performed within 28 days before the case.

★: If the administration of esvan or oxaliplatin is interrupted due to intolerable toxicity, the subject must conduct a toxicity assessment at least once a week to determine whether the re-administration can be continued.

Group C:

|  | Case | | Neoadjuvant chemotherapy | | | Surgery |
| --- | --- | --- | --- | --- | --- | --- |
|  |  |  | (Repeat 2 cycles) | | |  |
| Detection time | -28 Day | -14 Day | Week 1 | Week 2 | Week 3 |  |
| Oxaliplatin |  |  | Day 1 |  |  |  |
| Capecitabine |  |  |  |  | Withdrawal |  |
| Tumor measurement | ⊙◎ |  |  | ●Every 6 weeks |  |  |
| Laboratory examination |  | ⊙ | ⊙ |  |  | ⊙ |
| Electrocardiogram | ⊙ |  | ◇ | ◇ | ◇ | ◇ |
| Chest x-ray | ⊙ |  |  | ●Every 6 weeks |  |  |
| Subjective / objective symptoms |  | ⊙ | ⊙ |  |  | ⊙ |
| Vital signs |  | ▲ | ⊙ |  |  | ⊙ |

⊙: Necessary

●: Execute every 6 weeks after entering the case

○: Execute before or before giving Eswan or Oxaliplatin.
◇: Test when needed.

▲: Weight and physical fitness are necessary measures.

◎: Tumor measurement must be performed within 28 days before the case.

★: If the administration of esvan or oxaliplatin is interrupted due to intolerable toxicity, the subject must conduct a toxicity assessment at least once a week to determine whether the re-administration can be continued.

Second stage: adjuvant chemotherapy

Group A:

|  | Surgery | Postoperative combined adjuvant chemotherapy | | | End of treatment |
| --- | --- | --- | --- | --- | --- |
|  |  | (Repeat 6 cycles) | | |  |
|  |  |  |  |  |  |
| Detection time |  | Week 1 | Week 2 | Week 3 | Track 5 year survival |
| Oxaliplatin |  | Day 1 |  |  |  |
| Eswan |  |  |  | Withdrawal |  |
| Tumor measurement |  |  | ◇ |  |  |
| Laboratory examination | ⊙ | ⊙○ | ★ |  | ⊙ |
| Electrocardiogram | ◇ | ◇ | ◇ | ◇ | ◇ |
| Chest x-ray |  |  | ●Every 6 weeks |  |  |
| Subjective / objective symptoms | ⊙ | ⊙ | ★ |  | ⊙ |
| Vital signs | ⊙ | ⊙▲ |  |  | ⊙▲ |

⊙: Necessary

●: Execute every 6 weeks after entering the case

○: Execute before or before giving Eswan or Oxaliplatin.
◇: Test when needed.

▲: Weight and physical fitness are necessary measures.

◎: Tumor measurement must be performed within 28 days before the case.

★: If the administration of esvan or oxaliplatin is interrupted due to intolerable toxicity, the subject must conduct a toxicity assessment at least once a week to determine whether the re-administration can be continued.

Group B:

|  | Surgery | Postoperative combined adjuvant chemotherapy | | | End of treatment |
| --- | --- | --- | --- | --- | --- |
|  |  | (Repeat 6 cycles) | | |  |
|  |  |  |  |  |  |
| Detection time |  | Week 1 | Week 2 | Week 3 | Track 5 year survival |
| Oxaliplatin |  | Day 1 |  |  |  |
| Eswan |  |  |  | Withdrawal |  |
| Tumor measurement |  |  | ◇ |  |  |
| Laboratory examination | ⊙ | ⊙○ | ★ |  | ⊙ |
| Electrocardiogram | ◇ | ◇ | ◇ | ◇ | ◇ |
| Chest x-ray |  |  | ●Every 6 weeks |  |  |
| Subjective / objective symptoms | ⊙ | ⊙ | ★ |  | ⊙ |
| Vital signs | ⊙ | ⊙▲ |  |  | ⊙▲ |

⊙: Necessary

●: Execute every 6 weeks after entering the case

○: Execute before or before giving Eswan or Oxaliplatin.
◇: Test when needed.

▲: Weight and physical fitness are necessary measures.

◎: Tumor measurement must be performed within 28 days before the case.

★: If the administration of esvan or oxaliplatin is interrupted due to intolerable toxicity, the subject must conduct a toxicity assessment at least once a week to determine whether the re-administration can be continued.

Group C:

|  | Surgery | Postoperative combined adjuvant chemotherapy | | | End of treatment |
| --- | --- | --- | --- | --- | --- |
|  |  | (Repeat 6 cycles) | | |  |
|  |  |  |  |  |  |
| Detection time |  | Week 1 | Week 2 | Week 3 | Track 5 year survival |
| Oxaliplatin |  | Day 1 |  |  |  |
| Capecitabine |  |  |  | Withdrawal |  |
| Tumor measurement |  |  | ◇ |  |  |
| Laboratory examination | ⊙ | ⊙○ | ★ |  | ⊙ |
| Electrocardiogram | ◇ | ◇ | ◇ | ◇ | ◇ |
| Chest x-ray |  |  | ●Every 6 weeks |  |  |
| Subjective / objective symptoms | ⊙ | ⊙ | ★ |  | ⊙ |
| Vital signs | ⊙ | ⊙▲ |  |  | ⊙▲ |

⊙: Necessary

●: Execute every 6 weeks after entering the case

○: Execute before or before giving Eswan or Oxaliplatin.
◇: Test when needed.

▲: Weight and physical fitness are necessary measures.

◎: Tumor measurement must be performed within 28 days before the case.

★: If the administration of esvan or oxaliplatin is interrupted due to intolerable toxicity, the subject must conduct a toxicity assessment at least once a week to determine whether the re-administration can be continued.

2.2 Study target population

The target population of the study is patients who can undergo surgical resection of gastric cancer. The specific selection criteria are as follows:

2.2.1 Standard constrain

Inclusion conditions：

1) The age is at least 18 years old.

2) Histopathological or cytological diagnosis of gastric cancer (including gastric adenocarcinoma of the cardia).

3) Have not received anti-tumor treatment such as chemotherapy and radiotherapy before

4) Behavioral ability ECOG ≦ 2.

5) Stage III and Stage IV tumors.

6) Those without distant organ transfer (M0).

7) Sign informed consent.

2.2.2 Exclusion criteria

1. Exclusion conditions:

1) Patients who are known to be allergic to Aswan, Capecitabine, Oxaliplatin and their accessories.

2) Patients with severe bone marrow suppression (which may worsen symptoms):

(a) Leukocytes are less than 4,000 / mm^3^.

(b) Neutrophils are less than 2,000 / mm^3^.

(c) Platelets are less than 100,000 / mm^3^.

3) Patients with severe renal dysfunction or liver dysfunction:

(a) Bilirubin is greater than twice the upper limit of normal.

(b) Alanine aminotransferase (ALT) and aspartate aminotransferase (AST) indexes are greater than normal in patients without liver metastasis

2.5 times the upper limit of the value, patients with liver metastases are greater than 5 times the upper limit of the normal value.

(c) Plasma creatinine is greater than twice the upper limit of normal.

4) Present patients with peripheral neuropathy.

5) Are using other fluorouracil anti-tumor drugs (including combination chemotherapy with these drugs) or fluorophore

Patients with pyrimidine.

6) Female patients during pregnancy or breastfeeding, those of childbearing age (including men) who do not want to take contraceptive measures.

7) People with uncontrollable mental illness may have an influence on the understanding and compliance of the plan.

8) Myocardial infarction occurred within 6 months, or existing severe / unstable angina and cardiac insufficiency.

9) Severe organic lesions of important organs, such as severe chronic obstructive pulmonary disease, interstitial pneumonia, and lung fibers

It may cause respiratory failure, renal insufficiency or glomerular sclerosis; uncontrolled diabetes.

10) Those with severe infections who need treatment.

11) Oral chemotherapy caused by dysphagia, active peptic ulcer, gastrointestinal bleeding and perforation

People who have difficulties with drugs.

2.3.3 Criteria for case exclusion

1) The patient's condition progresses or recurs during treatment;

2) The patient withdraws the treatment consent;

3) The patient has a toxic and side reaction that the attending physician believes is necessary to terminate the trial treatment;

4) The patient has a serious concurrent disease that the attending physician believes is necessary to terminate the trial treatment;

5) The attending physician believes that the best option for the benefit of the patient is to terminate the trial treatment.3. Observation items and evaluation

1 Evaluation of efficacy

1.1 Evaluation of efficacy

The first stage: R0 removal rate

. Eswan combined with oxaliplatin is superior to the surgical group

. Capecitabine combined with oxaliplatin is not worse than Eswan combined with oxaliplatin

Stage 2: Disease-free survival (DFS)

. Eswan combined with oxaliplatin perioperative chemotherapy is superior to adjuvant chemotherapy alone

. Capecitabine combined with oxaliplatin is not worse than adjuvant therapy with Aswan combined with oxaliplatin

Secondary goals:

  Overall response rate (ORR), disease control rate (DCR)

  Downstaging Rate

  Overall survival (OS)

  Compliance of Aswan, Capecitabine, Oxaliplatin

  Safety

2 Safety evaluation

2.1 Evaluation of toxic and side effects

Observe the frequency of the main toxic and side effects of each medication course, and the attending physician comprehensively judges the adverse reaction and Verify the relationship between drugs, divided into: (1) definitely related; (2) may be related; (3) may not be related; (4) certainly not Off; (5) Unable to assess.

4. Aiswan dosage and adjustment

For the convenience of administration, the baseline amount for initial administration is recommended to be divided into 3 dosage levels according to the patient's body surface area . The details are as follows: Body surface area <1.25m2, the total daily dose of Eswan capsules is 80mg, that is, 40mg per oral administration, twice a day, orally after breakfast and after dinner; body surface area is ≥1.25 m2 ~ <1.5m2 , The total daily dose of Aiswan capsules is 100mg, namely 60mg orally after breakfast and 40mg orally after dinner; the body surface area is ≥1.5 m2, the total daily dose of Aiswan capsules is 120mg, that is 60mg per oral, 2 daily Oral after breakfast and after dinner. If the patient misses Eswan for any reason or vomits after taking the medicine, no supplement is needed. The physician can appropriately increase or decrease the dosage according to the state of the patient, and the dosage is set to 40, 50, 60, 75 mg / time. When the dose needs to be increased, if there are no abnormal clinical examination values (hematological examination, liver and kidney function test) related to Aiswan capsule and gastrointestinal symptoms, there is no safety problem, one dose can be increased in sequence according to the benchmark amount The maximum should not exceed 75mg / time; when reducing the dose, reduce one dose in sequence according to the reference amount, and the minimum dose is 40mg / time.

Refer to the table below when increasing or decreasing the dose：

| decreasing the dose | Baseline dose for initial administration | increasing |
| --- | --- | --- |
| Withdrawal | 40mg/time | 50mg/time |
| Withdrawal←40mg/time | 50mg/time | 60mg/time |
| Withdrawal←40mg/time←50mg/time | 60mg/time | 75mg/time |

Note: The above doses are based on tegafur. When increasing or decreasing the dose, it should be increased or decreased in accordance with the above table in each cycle.

5. Statistical analysis plan

The main purpose of this plan is to confirm that in the R0 resection rate, Eswan combined with oxaliplatin is superior to the surgical group,

α = 0.05, β = 0.2, the expected efficacy is 10%, capecitabine combined with oliplatin is not inferior to aswan combined with oxaliplatin. The sample size is estimated to be 656 people. Considering the 10% exclusion rate, a total of 729 subjects need to be included to achieve 80% test power.

Efficacy analysis: To compare the similarities and differences of different treatment methods by survival analysis and post-test.

Safety analysis: mainly based on descriptive statistical analysis, the list describes the toxic and side effects of this test.

6. References

[1] GLOBOCAN 2008 (IARC) Section of Cancer Information (12/4/2011)

[2]秦叔逵，龚新雷．晚期胃癌化疗的现状和进展．临床肿瘤学杂志，2006，11（9）：641-652．

[3]Shirasaka T,Nakano K,Takechi T,et al. Antitumor activity of S-1 against human colon carcinoma orthotopically implanted into nude rats.Cancer Res,1996,56(11):2602-2606.

[4] Taguchi T, Inuyama Y, Kanamaru R,et al.Phase I study of S-1. S-1 Study Group. Gan To Kagaku Ryoho. 1997,24(15):2253-2264.

[5] Hoff PM, Saad ED, Ajani JA,et al.Phase I Study with Pharmacokinetics of S-1 on an Oral Daily Schedule for 28 Days in Patients with Solid Tumors. Clin Cancer Res,2003,134(9): 134–142.

[6] Chu QS, Hammond LA, Schwartz G,,et al.Phase I and Pharmacokinetic Study of the Oral Fluoropyrimidine S-1 on a Once-Daily-for-28-Day Schedule in Patients with

Advanced Malignancies. Clin Cancer Res, 2004,10(15), 4913–4921.

[7] Koizumi W, Kurihara M, Nakano S, et al.Phase II study of S-1, a novel oral derivative of 5-fluorouracil, in advanced gastric cancer. For the S-1 Cooperative Gastric Cancer Study Group. Oncology. 2000,58 (3):191-197.

[8] Sakata Y, Ohtsu A, Horikoshi N, et al.Late phase II study of novel oral fluoropyrimidine anticancer drug S-1 in advanced gastric cancer patients. Eur J Cancer,1998,34(11):1715-1720.

[9] Koizumi W, Tanabe S, Saigenji K,et al. Phase I/II study of S-1 combined with cisplatin in patients with advanced gastric cancer. Br J Cancer,2003,89(12): 2207–2212.

[10] Sato Y, Kondo H, Honda K,et al.A phase I/II study of S-1 plus cisplatin in patients with advanced gastric cancer:2-week S-1 administration regimen. Int J Clin Oncol, 2005,10(1):40–44.

[11].Ajani JA, Faust J, Ikeda K,et al. Phase I Pharmacokinetic Study of S-1 Plus Cisplatin in Patients With Advanced Gastric Carcinoma. J Clin Oncol,2005, 23(28):6957-6965.

[12] Lee JL, Kang HJ, Kang YK,et al.Phase I/II study of 3-week combination of S-1 and cisplatin chemotherapy for metastatic or recurrent gastric cancer.Cancer Chemother Pharmacol, 2008 ,61(5):837-845.

[13].Boku N,Yamamoto S,Shirao K,et al.Randomized phase III study of 5-fluorouracil (5-FU) alone versus combination of irinotecan and cisplatin (CP) versus S-1 alone in advanced gastric cancer (JCOG9912) J Clin Oncol,2007,25(18S): LBA4513.

[14] Koizumi W,Narahara H, Hara T,et al.S-1 plus cisplatin versus S-1 alone for first-line treatment of advanced gastric cancer (SPIRITS trial): a phase III trial.Lancet Oncol,2008, 9(3): 215–221.
[15] Sakuramoto S,Sasako M,Yamaguchi T，et al.Adjuvant Chemotherapy for Gastric Cancer with S-1, an Oral Fluoropyrimidine.N Engl J Med,2007,357(18):181

7. Accessories:

Attachment 1: Physical condition scoring criteria Eastern Oncology Collaborative Group (ECOG) physical condition scoring criteria:

0 Ability to move is completely normal, and there is no difference from the ability to move before onset;

1 Can move freely and engage in light physical activities, including general housework or office work, but not engage in heavy physical activities;

2 Can walk freely and take care of himself, but he has lost his ability to work, and can get up at least half of the time during the day;

3 Life can only be partly self-care, bed or wheelchair for more than half of the day;

4 Bedridden, unable to take care of themselves;

5 Death.

Attachment 2: Evaluation Criteria for Objective Curative Effects of Cancer Drugs

Evaluation Criteria for Therapeutic Efficacy-RECIST Version 1.1 Evaluation Method

1. Basic Concepts

Only those patients with tumors that have measurable lesions at baseline can be selected for the trial. The tumor treatment efficacy of these patients is the main research purpose of clinical trials.

1. Measurable lesions-lesions that can be accurately measured on at least one diameter line (recorded as the largest diameter). Under normal testing conditions, the maximum diameter of the tumor should be "20mm," and the maximum diameter of the tumor should be 10mm during spiral CT detection. Lymph nodes with a short diameter of 15 mm are considered as measurable lesions.

2. Non-measureable lesions-other lesions, including small lesions (maximum diameter less than 20 mm under conventional testing conditions, or maximum diameter less than 10 mm during spiral CT) and truly non-measureable lesions. Lymph nodes with a short diameter between 10 and 15 mm are considered pathologically unmeasured non-target lesions.

3. Really unmeasured lesions-including the following: skeletal lesions, pial meningeal lesions, ascites, pleural / pericardial effusion, cutaneous lymphangiitis, abdominal masses that have not been confirmed or cannot be followed up by imaging, and cystic lesions. All measurement data is measured and recorded using a ruler or caliper, and the recorded results are expressed in metric metric system. All benchmark measurements should be completed as close as possible to the start of treatment, but not allowed to be completed before 4 weeks of treatment.

4. The number of target lesions-the maximum number of measurable target lesions used for judging the curative effect is 5 and 2 for each organ.

Second, the specific measurement method

For each identified and reported lesion, the same measurement technique and evaluation method should be used at baseline and at follow-up. If imaging methods and clinical examinations are used to evaluate antitumor efficacy, the former is superior to the latter.

1. Clinical examination Only when the lesion is located at the superficial site, such as skin nodules and palpable lymph nodes, can clinically detected lesions be considered measurable. For skin lesions, it is recommended to use color photos to record and archive, and the photos should have a scale to measure the size.

2. X-ray chest radiograph X-ray chest radiograph shows that the lesion located at the boundary of the lung parenchyma clearly can be accepted as a measurable lesion.

3. CT and MRI are currently used as the most reliable and reproducible methods for evaluating the efficacy of target lesions. Conventional CT and MRI are completed in a continuous scan with a layer thickness of 10 mm. Spiral CT can be completed in a continuous reconstruction mode with a layer thickness of 5 mm. CT is currently the most commonly used curative effect evaluation method and reproducible anatomical imaging technology in RECIST. The lesions evaluated by CT scanning must be measured at the same window, the baseline measurement of CT and MRI lesions and subsequent strategies must be On the same anatomical plane, use the same machine for scanning whenever possible. Unless there are contraindications such as allergies, contrast enhancement of veins should generally be given to distinguish blood vessels and soft tissue from adjacent tumor tissue.

4. B-mode ultrasound is not recommended for curative effect evaluation due to poor reproducibility, independent operation, and subjective results. But for superficial lymph nodes and subcutaneous lesions, ultrasound can be used as an alternative method.

5. Cytology When the measurable lesion is stable or effective, any exudate that appears and deteriorates during treatment requires cytology to confirm its tumor properties. Histology and cytology can be used to identify the nature of exudative lesions, that is, to determine whether exudation is caused by disease progression.

6. Endoscopy is used only to confirm the recurrence of the lesion after complete remission.

7. Serum or pleural and ascites tumor markers cannot be used as a basis for evaluating the efficacy.

3. Evaluation of the efficacy of cancer treatment

1. Baseline assessment

1) Evaluation of total tumor burden and measurable diseases

In order to evaluate the objective efficacy, the total tumor load at baseline was evaluated for comparison with subsequent measurements.

2) Baseline status records of target and non-target lesions

Calculate the sum of the longest diameter of all target lesions, and call it the sum of the longest diameter of the baseline state.

2. Efficacy evaluation

1) Evaluation of target lesions

Measure the longest diameter of all target lesions and calculate the sum of the longest diameter of all target lesions. Compared with the sum of the longest diameter of the baseline state, the evaluation criteria of tumor objective efficacy are as follows:

Complete Remission (CR): all target lesions disappear;

Partial Remission (PR): The sum of the longest diameters of target lesions is at least 30% lower than that of baseline;

Progression (PD): the sum of the longest diameters of target lesions is 20% higher than the baseline state, or the absolute value is increased by 5mm, or new lesions appear;

Stability (SD): between partial remission and stability.

2) Evaluation of non-target lesions:

Complete remission (CR): all non-target lesions disappear and tumor markers are normal;

Incomplete remission or stabilization (IR / SD): the presence of one or more non-target lesions and / or tumor markers persists above normal values;

Progression (PD): One or more new lesions appear, or obvious progress of existing non-target lesions.

3) Evaluation of the best total efficacy

The best total therapeutic effect is the minimum value measured from the beginning of treatment to disease progression or recurrence. In general, the classification of the patient's best curative effect consists of lesion measurement and confirmation.

Table 1 The total curative effect of all possible combinations of target and non-target lesions and new lesions

| Target lesion | Non-target lesion | New lesion | Total efficacy |
| --- | --- | --- | --- |
| CR | CR | No | CR |
| CR | Non CR/SD | No | PR |
| PR | No PD | No | PR |
| SD | No PD | No | SD |
| PD | Any | Yes/No | PD |
| Any | PD | Yes/No | PD |
| Any | Any | Yes | PD |

4) Symptomatic deterioration Patients with treatment discontinuation due to generalized health deterioration, although there is no evidence of PD, should be classified as "symptomatic deterioration", and even after interruption of treatment, they should do their best to record objective disease progression.

5) The conditions for early progression, early death, and inevitable events vary from experiment to experiment, and should be clearly defined in each protocol (depending on treatment time and treatment cycle).

6) In the case where it is difficult to distinguish between tumor and normal tissue, when it is evaluated as complete remission, it is recommended to perform fine needle aspiration / biopsy to confirm the suspected residual lesion before confirming complete remission.

3. Frequency of tumor reassessment

The frequency of tumor re-evaluation varies depending on the treatment plan. It should be a model that matches the treatment schedule. It is reasonable to re-evaluate every 2 cycles (6-8 weeks), or it is usually twice the treatment time. In the case of, it should be adjusted to a shorter or longer time.

4. Confirmation of measurement and duration of efficacy

1) The purpose of confirming objective efficacy confirmation is to avoid overestimation of efficiency. Changes in CR and PR tumor measurements must be repeatedly judged and confirmed, and must be reviewed and confirmed not less than 4 weeks after the first evaluation.

2) Remission period (total duration of efficacy)

It is the time interval from the first measurement of CR or PR until the first disease recurrence or progression.

3) Duration of stable disease

The time interval from the beginning of treatment to the progression of the disease, including the time from CR / PR / SD.

5. Results report

All patients in the trial, including those who deviated from the treatment plan or failed, must judge the efficacy of treatment (Intend to treatment, ITT), and each patient must be classified as follows (1) CR, (2) PR, (3) SD, (4) PD, (5) early death due to tumor, (6) early death due to toxicity, (7) early death due to other causes, (8) unclassified (not enough data to evaluate) All patients who meet the enrollment criteria should be included in the effective analysis, and all PDs and deaths should be considered as treatment failure. After excluding those who violate the main treatment plan (such as early death due to other reasons, early interruption of treatment, unfinished main treatment, etc.), subgroups of patients can be analyzed. However, conclusions about treatment effects cannot be drawn from the subgroup analysis, and the reasons for excluding the patient from the analysis must be clearly reported, and a 95% confidence interval must be provided.

Appendix 3: Commonly used toxicity classification standards

Part I Common Terminology Standards for Adverse Reactions (CTC AE) Version 4.0 Classification Standard

Toxicity judgment standards for some important organs

1. CTC AE classification, divided into 6 levels, including 0 ~ 5 levels

0 No adverse events occurred

1 Mild adverse events

2 Moderate adverse events

3 Serious adverse events

4 Life-threatening or disabling adverse events

5 death

Part 2 Safety Evaluation Method

This project specifies the definition of adverse events, the criteria for judging adverse events, the criteria for judging the relationship between adverse events and trials, the judgment of serious adverse events, and the reporting system for adverse events, using the "Common Terminology Standard (CTC-AE) version 3.0 "Requirements for safety assessment, see Annex IV for details.

(1) Definition of adverse events: From the beginning of the patient ’s signing of informed consent and being included in the trial, to the last follow-up, any adverse medical events that occur, regardless of whether there is a causal relationship with the test drug, are determined as adverse events. During the test, the adverse events were faithfully recorded, including the time, severity, duration, measures and outcomes of the adverse events.

(2) Judgment criteria for the severity of adverse events: refer to the commonly used toxicity classification standard CTC 3.0.

 Mild: does not affect the normal function of the subject;

 Moderate: affects the normal function of the subject to a certain extent;

 Severe: Obviously affect the normal function of the subject.

(3) Criteria for judging the relationship between adverse events and trials: Researchers should evaluate the possible associations between adverse events and test drugs, and refer to the following criteria:

 Definitely related: the time when the reaction occurred was in accordance with the chronological order of the medication, the response was in accordance with the known reaction type of the test drug, it was improved after the drug was stopped, and repeated administration appeared again.

 It may be related: the time when the reaction occurs corresponds to the chronological order of the medication, the response conforms to the known type of response of the test drug, and the patient's clinical status or other treatment methods may also produce the response.

 It may be irrelevant: the time of the reaction does not meet the chronological order of the medication, the response is not in line with the known type of reaction of the test drug, and the patient's clinical status or other treatment methods may also produce the reaction.

 Irrelevant: The time of the reaction does not conform to the chronological order of the medication. The reaction conforms to the known type of reaction of the non-experimental drug. The patient's clinical status or other treatment methods may also produce the reaction. The disease state improves or the response to other treatment methods is eliminated. Repeated use of other treatment methods appears.

 Unable to determine: The time when the reaction occurred has no clear relationship with the time sequence of the medication. The reaction is similar to the known reaction type of the test drug, and other drugs used at the same time may also cause the same reaction.

(4) Determination of serious adverse events (SAE):

 death

 life threatening

 Lead to hospitalization or prolonged hospitalization

 Permanent or severe disability

 Congenital malformations and defects

(5) Serious adverse event reporting system

Any serious adverse events that occurred during the clinical trial or within 30 days of the last treatment, regardless of whether they are related to the drug, should be immediately completed by telephone within 24 hours, and the serious adverse event form should be filled out within 7 days to report the plan The project leader (PI) of the team leader unit, the person in charge of clinical bidding, and the ethics committee.

Appendix 4: Tumor staging standards

Japanese classi ﬁ cation of gastric carcinoma: 3rd English edition

AJCC classi ﬁ cation of gastric carcinoma: 2010 VERSION 7.0

Appendix 5: Instructions for patients outside the hospital

Notes outside the hospital

1. Patient medication should be strictly in accordance with our requirements.

2. Please carefully record the patient's condition according to the observation form, so that we can fully understand and evaluate.

3. In the following cases, please record and contact us in time:

* Appetite decreased significantly

* Vomiting is greater than 2 times every 24 hours

* Increase in stool frequency more than two times, or the shape of stool or abdominal pain

* Painful erythema edema or ulcer

* Significant rash

*heat

* Severe numbness or loss of sensation or taste disturbance

* Hand or foot skin color changes or pain

* Other obvious discomfort

Annex VI: Center Number

Test center number:

01 Hebei Cancer Hospital

02 Shanxi Cancer Hospital

03 Shanxi Changzhi Heping Hospital

04 Inner Mongolia Autonomous Region Hospital

05 Affiliated Hospital of Inner Mongolia Medical College

06 Yongnian County Hospital

07 Anyang Cancer Hospital

08 Xingtai City People's Hospital

09 Cangzhou Central Hospital

10 Handan First Hospital

11 Qinhuangdao First Hospital

12 Hengshui Harison International Peace Hospital
